# The Human Transient Receptor Potential Melastatin 2 Ion Channel Modulates ROS Through Nrf2

**DOI:** 10.1038/s41598-019-50661-8

**Published:** 2019-10-01

**Authors:** Lei Bao, Fernanda Festa, Christopher S. Freet, John P. Lee, Iwona M. Hirschler-Laszkiewicz, Shu-jen Chen, Kerry A. Keefer, Hong-Gang Wang, Andrew D. Patterson, Joseph Y. Cheung, Barbara A. Miller

**Affiliations:** 1 Departments of Pediatrics, The Pennsylvania State University College of Medicine, P.O. Box 850, Hershey, Pennsylvania 17033 USA; 2 Departments of Biochemistry and Molecular Biology, The Pennsylvania State University College of Medicine, P.O. Box 850, Hershey, Pennsylvania 17033 USA; 3 Departments of Phamacology, The Pennsylvania State University College of Medicine, P.O. Box 850, Hershey, Pennsylvania 17033 USA; 4 Department of Molecular Toxicology, The Pennsylvania State University, University Park, Pennsylvania, USA; 5 The Center of Translational Medicine, Lewis Katz School of Medicine, Temple University, Philadelphia, Pennsylvania 19140 USA; 6 Department of Medicine, Lewis Katz School of Medicine, Temple University, Philadelphia, Pennsylvania 19140 USA

**Keywords:** Cancer metabolism, Paediatric cancer, Calcium signalling, Ion channel signalling

## Abstract

Transient receptor potential melastatin channel subfamily member 2 (TRPM2) has an essential role in protecting cell viability through modulation of oxidative stress. TRPM2 is highly expressed in cancer. When TRPM2 is inhibited, mitochondria are dysfunctional, ROS levels are increased, and cell viability is reduced. Here, the importance of NF-E2-related factor (Nrf2) in TRPM2-mediated suppression of oxidant stress was explored. In TRPM2 depleted cells, antioxidant cofactors glutathione, NADPH, and NADH were significantly reduced. Cytoplasmic and nuclear expression of Nrf2 and of IQGAP1, a modulator of Nrf2 stability regulated by intracellular calcium, were decreased. Antioxidant enzymes transcriptionally regulated by Nrf2 and involved in GSH, NADPH, and NADH generation were significantly lower including PRX1 and PRX3, GPX4, GSTP1, GCLC, and MTHFD2. The glutamine pathway leading to GSH production was suppressed, and ATP and GTP levels were impaired. Reconstitution with wild type TRPM2 or Nrf2, but not TRPM2 pore mutant E960D, rescued expression of enzymes downstream of Nrf2 and restored GSH and GTP. Cell viability, ROS, NADPH, NADH, and ATP levels were fully rescued by TRPM2 and partially by Nrf2. These data show that TRPM2 maintains cell survival following oxidative stress through modulation of antioxidant pathways and cofactors regulated by Nrf2.

## Introduction

Oxidative stress resulting from increased production of reactive oxygen species (ROS) is found in many cancers. ROS themselves activate cellular signals, molecules, and functions^1^. Whereas low levels of ROS can modulate cellular survival and metabolic pathways to enhance cell proliferation, cell death pathways are activated as ROS levels rise and damage DNA, proteins, and lipids^2,3^. To protect themselves from cytotoxic levels of ROS, cancer cells generally increase their antioxidant capacity. The transcription factor nuclear factor (erythroid-derived 2)-related factor-2 (Nrf2) regulates expression of greater than 200 genes, many of these antioxidant enzymes^4,5^. It regulates key components of the antioxidant response including enzymes involved in glutathione (GSH) and NADPH regeneration. Nrf2 is highly expressed in many malignant cells, protecting them from oxidative stress and chemotherapy, which can increase ROS above a cytotoxic threshold and cause irreversible cellular damage^6,7^.

TRPM2 belongs to the transient receptor potential (TRP) ion channel superfamily. Members of this superfamily, particularly the TRPM (Melastatin) subfamily, are involved in many fundamental cell functions including modulation of cell proliferation and survival^8–10^. TRPM2 was the second member of the TRPM family to be cloned and is expressed in many types of cells. It mediates entry of cations including Ca^2+^ into the cell^11,12^. Oxidative stress, TNFα, and amyloid β-peptide have been shown to activate the channel through production of ADP-ribose, which binds to the TRPM2 C-terminus^13–15^. TRPM2 is highly expressed in many cancers including melanoma^16^, breast cancer^17^, prostate cancer^18^, tongue cancer^19^, and neuroblastoma^20^, suggesting it promotes cell survival^21^. In neuroblastoma, TRPM2 has been shown to protect cell viability by maintaining mitochondrial function, cellular bioenergetics, autophagy and reducing ROS levels^20,22,23^. Inhibition of TRPM2 increases sensitivity of neuroblastoma to doxorubicin, and enhances cell death in a number of malignancies including T cell leukemia^24^, gastric cancer^25^, and triple-negative and estrogen-receptor positive breast cancer cell lines^21,26^. In many non-cancer models, TRPM2 has also been shown to preserve cell viability; for example, it protects the hearts of mice from ischemia/reperfusion injury^27–29^. In a subset of Guamanian amyotrophic lateral sclerosis and Parkinson dementia patients, a TRPM2 mutant (P1018L) was found which inactivates after channel opening, limiting Ca^2+^ entry and supporting the conclusion that TRPM2 is necessary for normal neuronal function^30^. However, in some models, primarily non-malignant, TRPM2 expression was alternatively shown to enhance cell death^31–33^; the mechanisms responsible for this difference are not known. The preponderance of data in cancer models support the concept that TRPM2 expression and function preserves cell viability.

TRPM2 inhibition in neuroblastoma reduces cell viability through mitochondrial dysfunction, decreased cellular bioenergetics, and increased ROS levels^22,23^. When increased ROS are produced, malignant cells increase antioxidant capacity to prevent tissue damage. A number of antioxidant pathways are involved in conversion of superoxide anions to H_2_O_2_ and then to water^2,34^. Among these, the Nrf2 transcription factor is critically important in regulating expression of antioxidant enzymes and cofactors to reduce excessive ROS levels found in tumor cells^5^. The IQ motif containing GTPase activating protein 1 (IQGAP1) plays a critical role in maintaining Nrf2 stability and modulating nuclear expression through a Ca^2+^ dependent process^35^. The Ketch-like ECH-associated protein 1 (Keap1) is also a key factor which targets Nrf2 for ubiquitination and proteasomal degradation. In cellular stress, the Nrf2-Keap1 interaction is disrupted, Nrf2 ubiquitination decreased, and Nrf2 levels increased^2,36^. Nrf2 induces expression of many antioxidant enzymes including perioxiredoxins (PRXs) and glutathione peroxidases (GPXs), which detoxify H_2_O_2_ to water, and NAD(P)H quinone oxidoreductase 1 (NQO1) and heme oxygenase 1 (HMOX1)^37,38^. Nrf2 is critical in generation of antioxidant cofactors including GSH, the most abundant antioxidant cofactor in the cell, NADPH, and NADH^2,34^. Perioxiredoxins and glutathione peroxidases require NADPH to convert H_2_O_2_ to water. GPXs oxidize reduced glutathione (GSH), and the oxidized form, glutathione disulfide (GSSG), is converted back to GSH to replenish the supply by NADPH and glutathione reductase (GR), also regulated by Nrf2. Glutathione (GSH) is produced by conversion of glutamine to glutamate by glutaminase (GLS), followed by conversion of glutamate to GSH by glutamate-cysteine ligase (GCL) and glutathione synthetase (GSS), also Nrf2 transcriptional targets^39^.

Here, the function of TRPM2 in modulation of oxidative stress in cancer was examined by exploring its role in generation of the antioxidant cofactors GSH and NAPDH, and regulation of expression of the transcription factor Nrf2. Neuroblastoma cell lines and xenografts in which TRPM2 was depleted with CRISPR were utilized. Major findings are: (1) TRPM2 function is necessary to maintain GSH, NADPH, and NADH levels, and when TRPM2 is inhibited, these antioxidant cofactors are significantly reduced; (2) TRPM2 modulates Nrf2 expression and that of downstream enzymes involved in GSH, NADPH, and NADH generation in protection from oxidative stress; (3) Nrf2 regulatory proteins IQGAP1and Keap1 are reduced in TRPM2 depleted cells; (4) in the TRPM2 knockout (KO), reduced expression of enzymes involved in generation of GSH and low levels of glutamine also contribute to reduced GSH; and (5) reconstitution with TRPM2 completely restored cell viability, Nrf2 expression, and GSH, NAD^+^/NADH, NADP^+^/NADPH, ATP, GTP, and glutamine levels, whereas the pore mutant E960D did not. ROS were reduced to levels found in controls by TRPM2 expression. Reconstitution with Nrf2 fully restored GSH and GTP, partially restored cell viability, ATP, NADH, and NADPH, and significantly reduced ROS. These studies demonstrate an important mechanism through which TRPM2 protects cell viability and modulates oxidative stress, through regulation of Nrf2.

## Results

### TRPM2 modulates levels of the antioxidant cofactors GSH, NADPH, and NADH

**Figure 1:**
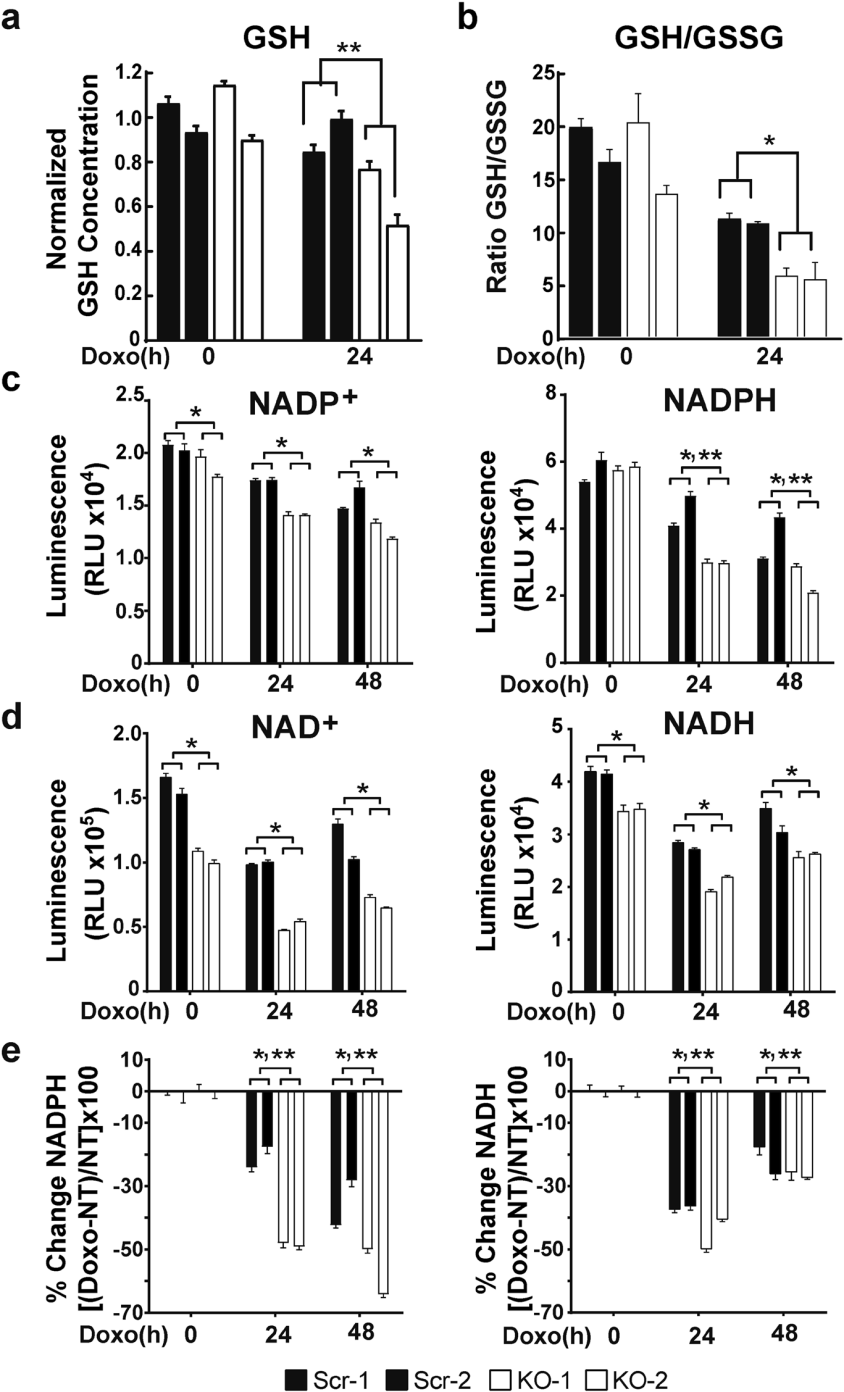
GSH, NADP^+^/NADPH, and NAD^+^/NADH levels are significantly reduced in TRPM2 depleted cells. (**a**,**b**) GSH concentration was measured in two clones of SH-SY5Y cells in which TRPM2 was depleted (KO-1 and KO-2) and in scrambled controls (Scr-1 and Scr-2). Cells were untreated or treated with 0.3 µM doxorubicin for 24 hours (Doxo 24 h). (**a**) In three experiments, the GSH concentration of each measurement was normalized to the average of each experiment’s untreated scrambled controls. Normalized means ± SEM are shown. **p < 0.0001, group x doxorubicin exposure time interaction effect, KO vs scrambled, two-way ANOVA. (**b**) Concentrations of GSH and GSSG were measured in triplicate and ratios calculated for each clone. A representative experiment of three is shown. *p < 0.01, group effect, two-way ANOVA. (**c**) NADP^+^ and NADPH or (**d**) NAD^+^ and NADH were measured in SH-SY5Y Scr or KO clones untreated or treated with 0.3 µM doxorubicin for 24 or 48 hours. Four experiments were performed and measurements from one representative experiment (n = 4 replicates) are shown. *p < 0.0001, group effect; **p < 0.0001, group x doxorubicin exposure time interaction effect, two way ANOVA. (**e**) Comparison of percent change in NADPH and NADH for each group [(NADPH or NADH in doxorubicin treated cells - untreated cells) divided by untreated cells, X 100%]. *p < 0.0001, group effect; **p < 0.03, group x doxorubicin exposure time interaction effect, two way ANOVA.

Superoxide anions are converted to H_2_O_2_ and water by enzymes which require the cofactors glutathione (GSH), NADPH and/or NADH. High ROS levels are found in TRPM2 depleted neuroblastoma cells compared to controls^20,22^, showing that maintenance of GSH and NADPH levels in these cells is critically important in regulating cytotoxicity. The role of TRPM2 in generation and reduction of these cofactors was examined in SH-SY5Y neuroblastoma cells following depletion of TRPM2 with CRISPR/Cas9 technology (KO). The GSH concentration in cells in which TRPM2 was depleted was significantly reduced compared to control cells after induction of oxidative stress with doxorubicin treatment. The decrease in mean ± SEM GSH concentration in three experiments, normalized to the average GSH concentration in scrambled control cells in each experiment, is shown in Fig. 1a. GSH levels in untreated TRPM2 depleted cells were not significantly different than control cells. The GSH/GSSG ratio in TRPM2 depletion was significantly less after doxorubicin than in scrambled control cells (Fig. 1b), consistent with reduced conversion of GSSG to GSH. These results demonstrate reduced GSH in TRPM2 depleted cells after oxidative stress and show that TRPM2 is required to maintain GSH generation.

NADPH and NADH are important cofactors for antioxidant enzymes which are involved in cytoplasmic and mitochondrial conversion of oxidized glutathione to GSH. The role of TRPM2 in generation and reduction of NADPH and NADH was examined in TRPM2 depleted SH-SY5Y cells and scrambled control clones. NADP^+^, NAD^+^, NADPH, and NADH were significantly reduced in TRPM2 depleted cells (p < 0.0001, group effect; Fig. 1c,d). For NADPH, there was significant group x doxorubicin exposure time interaction effect (p < 0.0001) between depleted and control cells (Fig. 1c), indicating doxorubicin exposure enhanced suppression of NADPH in TRPM2 depleted cells. The percent decrease in NADPH and NADH at 24 and 48 hours after doxorubicin was significantly greater in TRPM2 depleted cells compared to scrambled controls (Fig. 1e). These results demonstrate that TRPM2 is required to maintain NADPH and NADH and that levels of these cofactors are reduced in TRPM2 depleted cells, particularly after oxidative stress.

### TRPM2 is an important modulator of Nrf2 expression and enzymes involved in the antioxidant response

**Figure 2:**
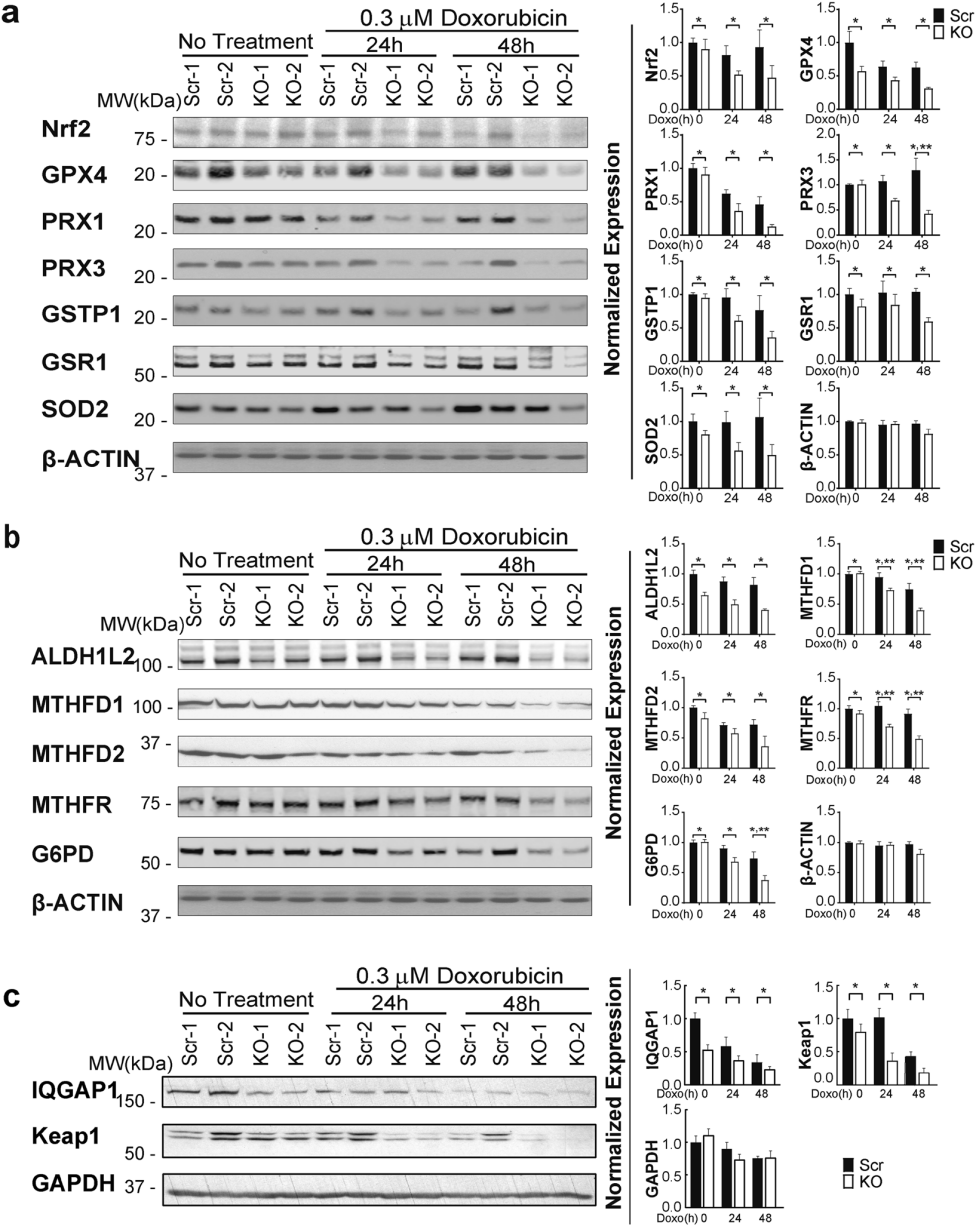
Nrf2, downstream antioxidant enzymes, and IQGAP1 are reduced in TRPM2 depleted cells following doxorubicin. SH-SY5Y Scr and TRPM2 KO clones were untreated or treated with 0.3 µM doxorubicin for 24 or 48 hours. Data from representative Western blots from three independent experiments are shown in (**a**–**c**). Intensify of bands was quantitated and measurements were normalized by comparing bands to each protein’s time 0 average scrambled control. Normalized means ± SEM for Scr and KO cells at each time point from three experiments are shown (n = 6 replicates) are shown to the right of the blots. (**a**) Western blotting was performed with antibodies to Nrf2, GPX4, PRX1, PRX3, GSTP1, GSR1, SOD2, and actin. p values analyzed with two-way ANOVA: Nrf2 (*p = 0.035), GPX4 (*p = 0.0002), PRX1 (*p = 0.0036), PRX3 (*p = 0.0002), GSTP1 (*p = 0.01), GSR1 (*p = 0.011), SOD2 (*p < 0.01), actin (p < 0.282), group effect; PRX3 (**p < 0.004), group x doxorubicin exposure time interaction effect. (**b**) Western blotting was performed with antibodies to ALDH1L2, MTHFD1, MTHFD2, MTHFR, and G6PD. p values analyzed with two-way ANOVA: ALDH1L2, MTHFD1, and MTHFR (*p < 0.0001), MTHFD2 (*p = 0.0135), G6PD (*p = 0.002), actin (p < 0.282), group effect; MTHFD1 and MTHFR (**p ≤ 0.02), G6PD (**p = 0.045), group x doxorubicin exposure time interaction effect. (**c**) Western blotting was performed with antibodies to IQGAP1, Keap1, and GAPDH. *p < 0.0014, group effect, two-way ANOVA.

**Figure 3:**
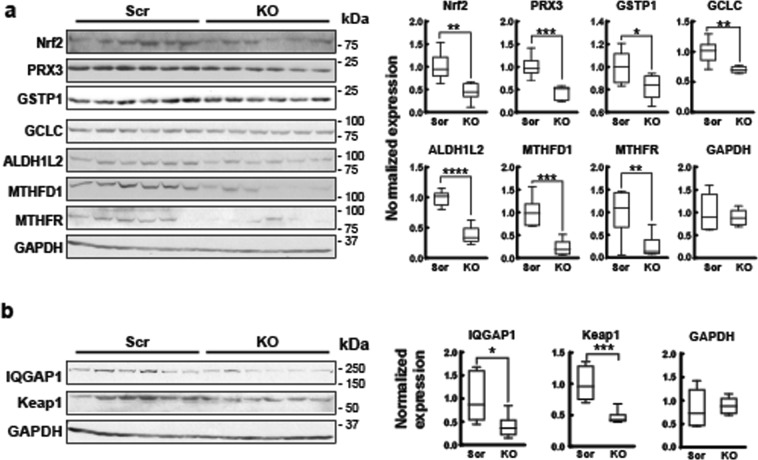
Nrf2, downstream antioxidant enzymes, and IQGAP1 are reduced in TRPM2 depleted xenografts. Xenografts were prepared by injecting flanks of Nude mice with TRPM2 depleted SH-SY5Y cells or scrambled controls. Tumors were harvested from six xenografts from KO cells and six from scrambled control cells after 6–7 weeks of growth, lysates prepared and Western blotting performed. (**a**) Western blotting was performed with antibodies to Nrf2, PRX3, GSTP1, GCLC, ALDH1L2, MTHFD1, MTHFR, and GAPDH. Intensify of bands was quantitated and KO results normalized to the mean of scrambled controls are shown on the right. Box plots with median, minimum, and maximal values are shown (n = 6 replicates/group). *indicates p < 0.05, ** < 0.01, *** < 0.001, **** < 0.0001, Student’s T-test. (**b**) Western blotting was performed with antibodies to IQGAP1, Keap1, and GAPDH and densitometry performed. Box plots with median, minimum, and maximal normalized values are shown (n = 6/group). IQGAP1 (*p < 0.03), Keap1 (***p < 0.001), Student’s T test.

Because GSH and NADPH production are compromised in TRPM2 depleted cells, the role of enzymes associated with the antioxidant response was examined. Nrf2, a master transcription factor which regulates expression of many antioxidant enzymes, was significantly reduced in TRPM2 depleted cells compared to controls after 24–48 hours of doxorubicin exposure (Fig. 2a). In xenograft tumors, Nrf2 was reduced in depleted cells even without treatment (Fig. 3a), possibly because of conditions of oxidative and metabolic stress existing within the tumor environment after weeks of tumor growth. Nrf2 regulates expression of the glutathione peroxidases (GPX4) and the perioxiredoxins (PRX1, PRX3), which convert H_2_O_2_ to water, and these enzymes were significantly decreased in TRPM2 depleted cells (Figs 2a and 3a). Glutathione-S-transferase P1 (GSTP1) catalyzes conjugation of reactive substrates to glutathione, reducing their toxicity and resulting in their eventual metabolism and exportation. It is also involved in control of the cell cycle^40^. GSTP1 is regulated by Nrf2 and was reduced in TRPM2 depleted cells (Figs 2a and 3a). Superoxide dismutase (SOD2) levels were also decreased (Fig. 2a). Enzymes involved in maintaining GSH levels were reduced. Glutathione reductase 1 (GSR1) catalyzes reduction of glutathione disulfide (GSSG) to glutathione, and is particularly important under oxidative stress. Glutamate-cysteine ligase catalytic subunit (GCLC) is involved in the conversion of glutamate to GSH. Expression of GSR1 and GCLC are regulated by Nrf2, and both GSR1 (Fig. 2a) and GCLC (Fig. 3a) were reduced in TRPM2 depleted cells.

NADPH and NADH function as cofactors for many antioxidant enzymes, donating reducing equivalents in conversion to NADP^+^ and NAD^+^. The enzymes aldehyde dehydrogenase 1 member L2 (ALDH1L2), and methylenetetrahydrofolate dehydrogenase (MTHFD1, MTHFD2) are involved in NADPH generation and are significantly decreased in TRPM2 depletion (Figs 2b and 3a), as is G6PD, which reduces NADP^ +^ to NADPH while oxidizing glucose-6-phosphate and is in the pentose phosphate pathway (Fig. 2b)^41^. Of these, MTHFD2 and G6PD have been shown to be regulated by Nrf2. MTHFR converts NADPH to NADP^+^ and is reduced in TRPM2 depletion (Fig. 2b and 3a). The decreases in MTHFD1, MTHFR, and G6PD were enhanced by doxorubicin exposure (Fig. 2b). These data demonstrate that Nrf2 and downstream enzymes involved in GSH and NADPH/NADH generation and reduction and key to antioxidant defense are decreased in TRPM2 depletion.

### IQGAP1 and Keap1 are decreased in TRPM2 depleted cells

IQGAP1 and Keap1 are proteins which post-translationally regulate Nrf2. IQGAP1 increases cytosolic and nuclear expression of Nrf2 and enhances Nrf2 translocation into the nucleus through a calcium dependent process^35^. IQGAP1 was significantly decreased in TRPM2 depleted cells cultured *in vitro* (Fig. 2c), and in xenografts (Fig. 3b). Keap1 is an important regulator of Nrf2 expression and facilitates Nrf2 ubiquitination^36^. Levels of Keap1 were decreased in TRPM2 depleted cells grown in culture (Fig. 2c) and in xenografts (3b). Decreased Keap1 would be predicted to increase Nrf2. However, the Nrf2 regulatory network is complex^5^, and these data suggest that other factors including reduced IQGAP predominate to lower Nrf2 levels in TRPM2 depletion^35,42,43^.

### Reduced glutamine contributes to decreased GSH levels in TRPM2 depleted cells

**Figure 4:**
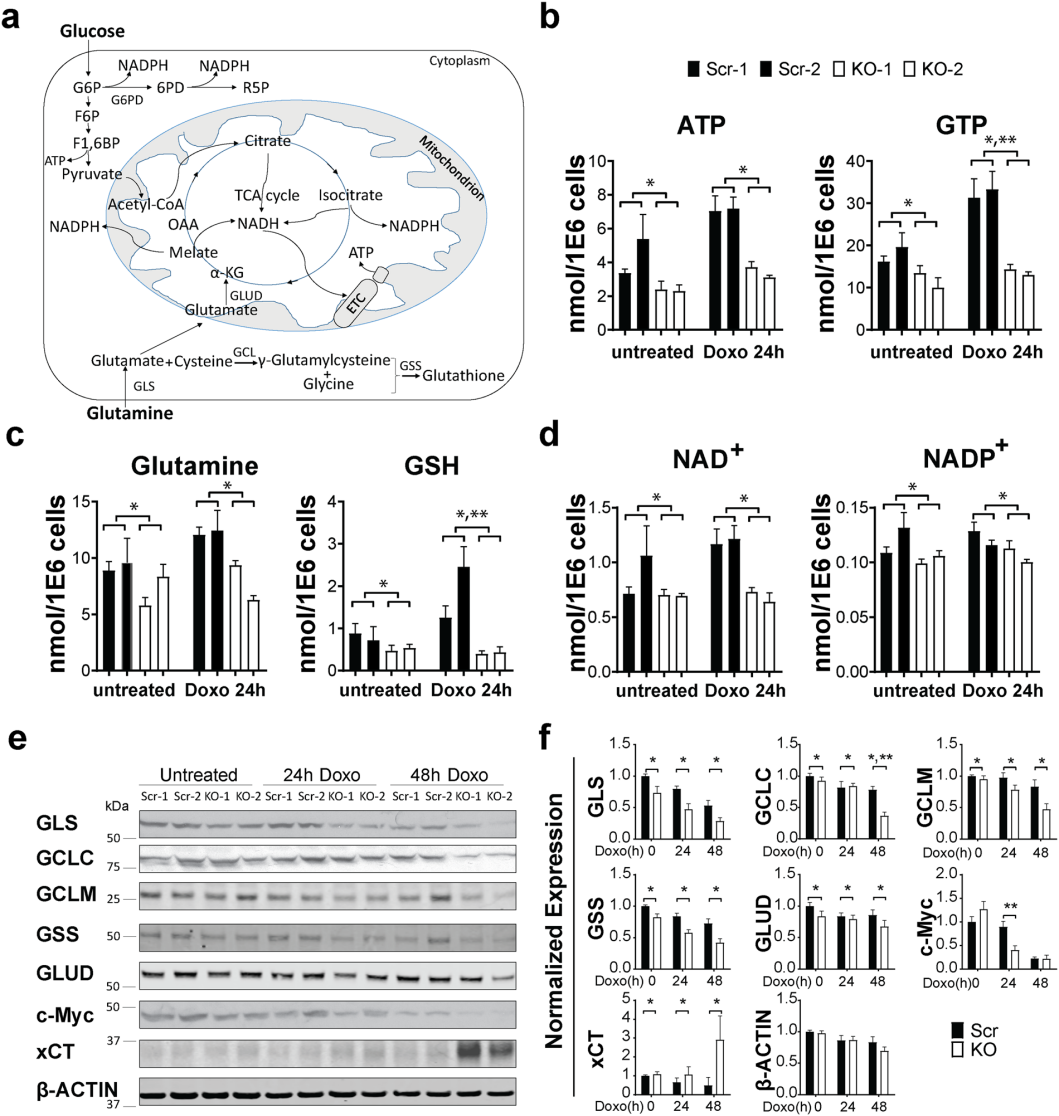
Decreased glutamine levels contribute to reduced GSH in TRPM2 depleted cells. (**a**) Schema of glutamine metabolism and GSH production. (**b**–**d**) Metabolomic quantitation of TRPM2 depleted and scrambled control cells treated with or without doxorubicin (n = 6 replicates/group) showed decreased (**b**) ATP and GTP, (**c**) glutamine and GSH, and (**d**) NAD^+^ and NADP^+^ in KO cells compared to scrambled control. *p ≤ 0.003, group effect; **p < 0.005, group x doxorubicin exposure time interaction effect, two way ANOVA. (**e**) Western blotting of proteins involved in GSH and α-ketoglutarate synthesis including GLS, GCLC, GCLM, GSS, GLUD, c-Myc, xCT, and β-actin. Representative blots from three experiments are shown. Densitometry measurements were normalized to the average of each blots’ untreated scrambled controls and mean densitometry measurements from the three experiments are shown in (**f**). p values analyzed with two-way ANOVA: GLS and GSS (*p < 0.0001), GCLC (*p < 0.007), GCLM (*p < 0.003), GLUD (*p < 0.04), xCT (*p < 0.05), group effect; GCLC (**p = 0.006), c-Myc (**p = 0.002), group x doxorubicin exposure time interaction effect.

Metabolomics analysis was used as a second approach to study the impact of TRPM2 depletion in cancer metabolism. Pathways involved in glutathione, NADPH, and NADH production and utilization are shown in Fig. 4a. TRPM2 depleted SH-SY5Y cells and scrambled control cells were untreated or treated with doxorubicin. Decreased levels of ATP, GTP, glutamine, GSH, NAD^+^, and NADP^+^ were confirmed with metabolomics analysis of TRPM2 depleted cells (Fig. 4b–d). NADH and NADPH were not measured in our metabolomic analysis. Doxorubicin treatment further decreased levels of GTP and GSH in TRPM2 depleted cells (group x doxorubicin exposure time interaction effect, p < 0.005, Fig. 4b,c).

Metabolism of glutamine has an important role in cellular bioenergetics, nucleotide synthesis, and ROS homeostasis through synthesis of glutathione^39^. Glutamine is transported into cells or acquired through breakdown of macromolecules including autophagy, and is converted to glutamate by glutaminase (GLS) (Fig. 4a). Glutamate can be converted to glutathione (GSH) by glutamate-cysteine ligase (GCL) and glutathione synthetase (GSS) or to α-ketoglutarate (α-KG) by glutamate dehydrogenase (GLUD) or aminotransferases; α-ketoglutarate enters the TCA cycle to produce ATP, NADPH, and NADH. Levels of the enzymes GLS, GCLC, glutamate-cysteine ligase modifier subunit (GCLM), GSS, and GLUD were all significantly decreased in TRPM2 depleted cells (Fig. 4e,f), supporting the conclusion that reduced synthesis contributes to decreased GSH in the TRPM2 KO. GLS, GCLC, GCLM, and GSS are downstream targets of Nrf2^38,44^ and GLS, GLUD and aminotransferases are targets of Myc^39,45^, a transcription factor which was also decreased in TRPM2 depleted cells (Fig. 4e,f). Of note, a representative glutamine transporter, xCT, was studied, and was significantly increased after doxorubicin in KO cells^46^, suggesting that reduced glutamine transport in the KO was not a contributing factor.

### Wild type TRPM2 but not the pore mutant E960D reconstitutes cell viability, GSH, NAD^+^/NADH, and NADP^+^/NADPH in TRPM2 depleted cells

**Figure 5:**
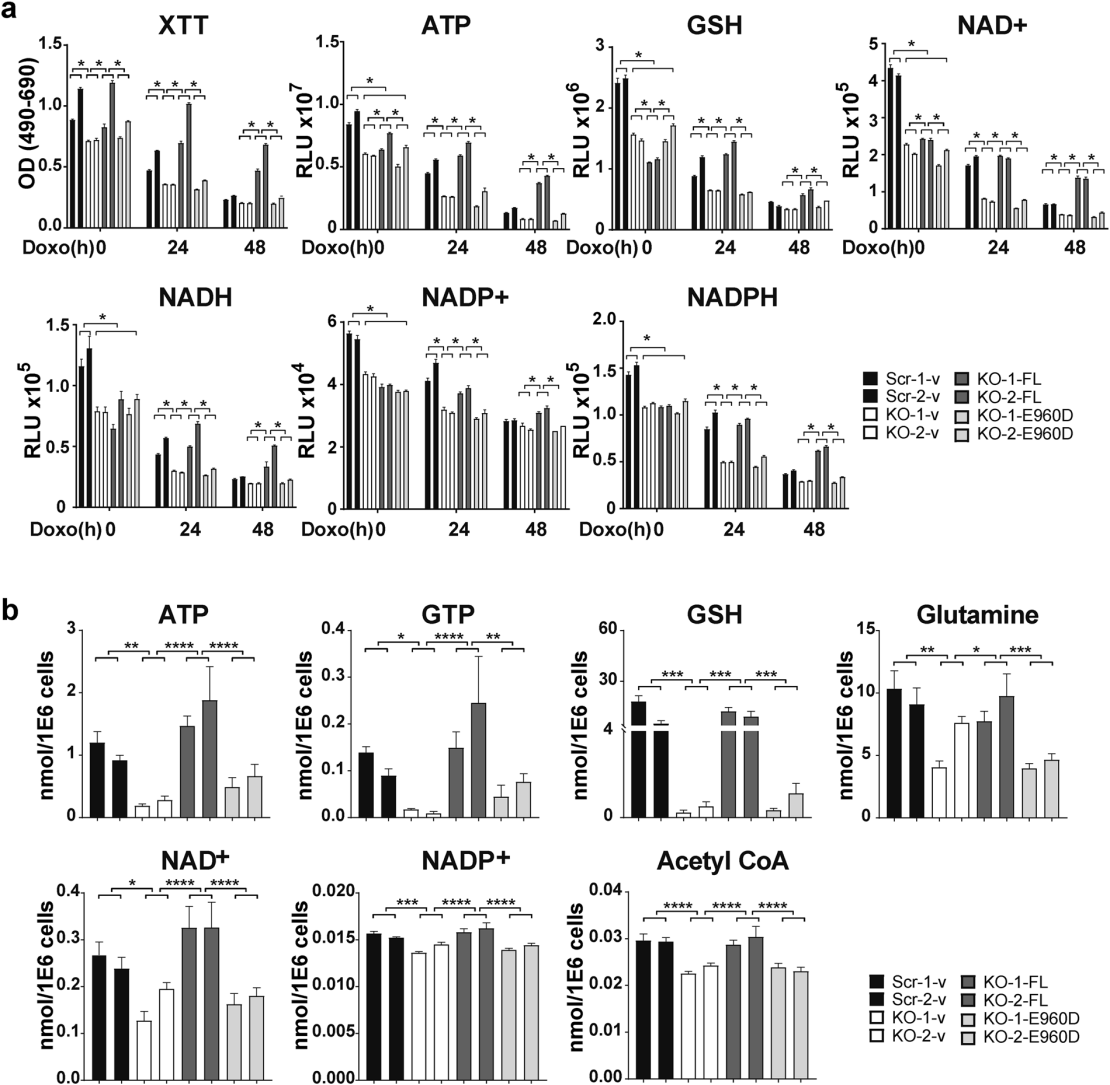
Wild Type TRPM2 but not the pore mutant E960D reconstitutes cell viability, ATP, GSH, NAD^+^, NADH, NADP^+^, and NADPH levels in TRPM2 depleted cells. SH-SY5Y cells in which TRPM2 was depleted (KO-1 and KO-2) were stably transfected with empty vector (KO-v), wild type TRPM2 (KO-FL), or the TRPM2 pore mutant E960D (KO-E960D). (**a**) Cells were untreated or treated with 0.3 µM doxorubicin for 24 or 48 hours. Cell viability was measured with XTT, and ATP, GSH, NAD^+^, NADH, NADP^+^, and NADPH levels were quantitated with biochemical assay kits. Four experiments were performed and a representative experiment is shown. *p < 0.003, was considered statistically significant using two-way ANOVA and the Bonferroni Correction. (**b**) Metabolomic analysis of TRPM2 depleted cells reconstituted with wild type TRPM2 or E960D and treated with 0.3 µM doxorubicin for 24 hours was performed (n = 6 replicates/group). ATP, GTP, GSH, glutamine, NAD^+^, NADP^+^, and Acetyl CoA were fully restored by expression of wild type TRPM2, but not with E960D. *p < 0.05, ** < 0.01, *** < 0.001, **** < 0.0001, one-way ANOVA.

To eliminate the possibility of off target effects occurring during TRPM2 depletion through CRISPR/Cas9, SH-SY5Y cells in which TRPM2 was depleted were stably transfected with empty vector, wild type TRPM2, or the TRPM2 calcium impermeable mutant E960D^47,48^. Scrambled control cells were transfected with empty vector. Cells were untreated or treated with 0.3 µM doxorubicin for 24 or 48 hours. The viability of TRPM2 depleted cells was restored to equal or greater than that of scrambled control cells by expression of wild type TRPM2 but not by E960D (Fig. 5a). By analysis with biochemical assay, wild type TRPM2 but not E960D restored ATP, GSH, NAD^+^, NADH, NADP^+^, and NADPH levels after doxorubicin to that of scrambled control cells (Fig. 5a). Metabolomic analysis confirmed that wild type TRPM2 restored GSH, NAD^+^, NADP^+^ as well as the metabolites ATP, GTP, glutamine, and Acetyl CoA (Fig, 5b).

**Figure 6:**
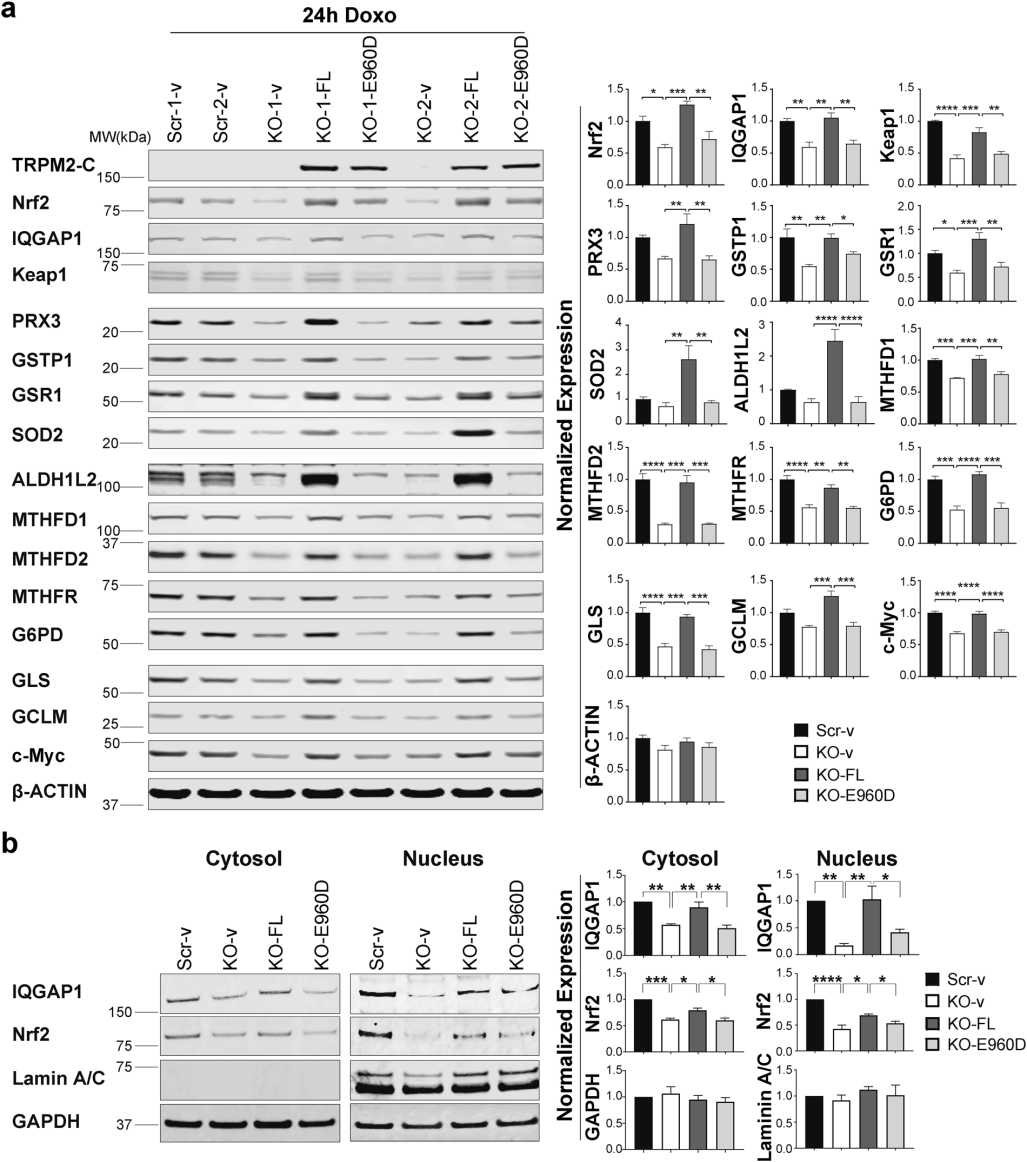
Expression of wild type TRPM2 but not E960D restores expression of Nrf2 and antioxidant enzymes, and Nrf2 nuclear translocation in TRPM2 depleted cells. SH-SY5Y TRPM2-KO cells were stably transfected with empty vector (KO-v), wild type TRPM2 (KO-FL), or E960D (KO-E960D). Scrambled controls were transfected with empty vector (Scr-v). Cells were treated with 0.3 µM doxorubicin for 24 hours. (**a**) Western blotting was performed with antibodies to TRPM2-C, Nrf2, proteins which regulate Nrf2 (IQGAP1, Keap1), antioxidant enzymes regulated by Nrf2 (PRX3, GSTP1, GSR1), SOD2, proteins involved in NADH and NADPH generation (ALDH1L2, MTHFD1, MTHFD2, MTHFR, G6PD), proteins involved in GSH synthesis (GLS, GCLM), c-Myc, and actin. Two experiments were performed and representative Western blots are shown. Intensify of bands was quantitated with Li-Cor technology. Blots were normalized by comparing bands to each protein’s average scrambled control. Normalized means ± SEM for each protein and group from two experiments are shown (n = 4). (**b**) Cells lysates were separated into cytoplasmic and nuclear fractions and Western blots were probed with antibodies to IQGAP1 and Nrf2. Lamin A/C (nuclear) and GAPDH (cytoplasmic and nuclear) were probed to demonstrate quality of fractionation. Representative Western blots from three experiments are shown. Mean ± SEM of densitometry measurements from the three normalized to Scr-v control is shown on the right (n = 3). For (**a**) and (**b**), *indicates p < 0.05, ** < 0.01, *** < 0.001, **** < 0.0001, one-way ANOVA.

Expression of transfected TRPM2 and the pore mutant E960D was confirmed with Western blotting (Fig. 6a). Endogenous expression of TRPM2 in scrambled cells is not seen in the exposure of the Western blot shown because of its relatively low expression level^22^. Transfection with wild type TRPM2 but not E960D restored expression of Nrf2 and downstream enzymes involved in GSH, NADPH, NADH, and glutamine production (Fig. 6a). The reduction in IQGAP1 and Keap1 in TRPM2 depleted cells was also restored by TRPM2 but not E960D expression. To examine the mechanisms through which TRPM2 modulates Nrf2, IQGAP1 and Nrf2 expression were examined in cytosolic and nuclear fractions of TRPM2 depleted cells. IQGAP1 and Nrf2 were significantly reduced in both fractions after doxorubicin, and decreased Nrf2 expression paralleled that of IQGAP1 (Fig. 6b). Reconstitution with TRPM2 but not E960D restored cytoplasmic and nuclear IQGAP1 and Nrf2. We examined mRNA levels of Nrf2 and IQGAP1 with RT-PCR. In TRPM2 depleted cells, no decrease in Nrf2 mRNA was observed in the KO (Supplemental Fig. S1) suggesting that the major mechanism for the decrease in Nrf2 was post transcriptional. In contrast, IQGAP1 mRNA was reduced in TRPM2 depleted cells (Supplemental Fig. S1). These results suggest that reduced expression of IQGAP1 together with lower intracellular calcium levels in TRPM2 depleted cells^22,35^ contribute to reduced stabilization and nuclear translocation of Nrf2 in the KO.

### Nrf2 fully reconstitutes GSH and partially restores cell viability and antioxidant cofactors in TRPM2 depletion

**Figure 7:**
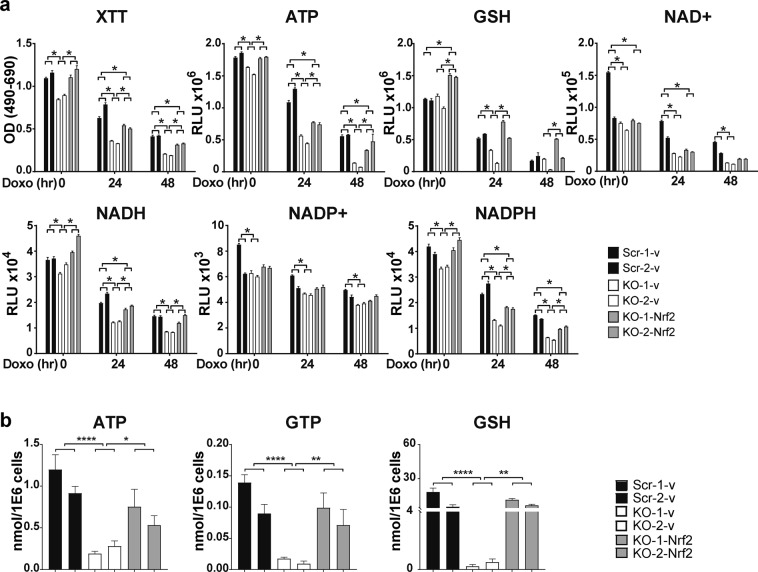
Reconstitution of Nrf2 fully restores GSH and GTP and partially restores cell viability, ATP, NADH, and NADPH levels in TRPM2 depleted cells. SH-SY5Y cells in which TRPM2 was depleted (KO-1 and KO-2) were stably transfected with Nrf2 (KO-Nrf2) or empty vector (KO-v), and scrambled controls cells with empty vector (Scr-v). (**a**) Cells were treated with media or 0.3 µM doxorubicin for 24 or 48 hours. Cell viability was measured with XTT, and ATP, GSH, NAD^+^, NADH, NADP^+^, and NADPH were quantitated with biochemical assays. Representative results from three experiments are shown. *p < 0.0056, significance determined with the Bonferroni Correction, two-way ANOVA. (**b**) Metabolomic analysis of TRPM2 depleted cells reconstituted with Nrf2 and treated with doxorubicin for 24 hours was performed (n = 6 replicates/group). Levels of ATP were partially restored, and GTP and GSH fully restored by expression of Nrf2. *p < 0.05, ** < 0.01, *** < 0.001, **** < 0.0001, one-way ANOVA.

To determine the importance of Nrf2 in TRPM2 function, SH-SY5Y cells in which TRPM2 was depleted were stably transfected with Nrf2. Cells were untreated or treated with 0.3 µM doxorubicin for 24 or 48 hours. Expression of transfected Nrf2 and downstream enzymes was confirmed by Western blotting (Supplemental Fig. S2). Nrf2 fully restored the proliferation of untreated TRPM2 depleted cells, and partially restored viability after doxorubicin treatment (Fig. 7a). GSH and GTP were fully restored by Nrf2 reconstitution, shown by biochemical assay (Fig. 7a) and metabolomic analysis (Fig. 7b) respectively. Nrf2 partially restored ATP (Fig. 7a,b), NADH (Fig. 7a), and NADPH (Fig. 7a). NAD^+^ and NADP^+^ levels were not significantly altered by Nrf2 reconstitution (Fig. 7a and metabolomic analysis, not shown). Full restoration of GLS and GCLC and partial restoration of other enzymes downstream of Nrf2 was demonstrated by Western blotting (Supplementary Fig. S2). These data demonstrate that TRPM2 maintains GSH, NADH, and NADPH levels during oxidative stress and that the mechanism involves modulation of Nrf2.

### ROS are reduced by reconstitution of TRPM2 and Nrf2

**Figure 8:**
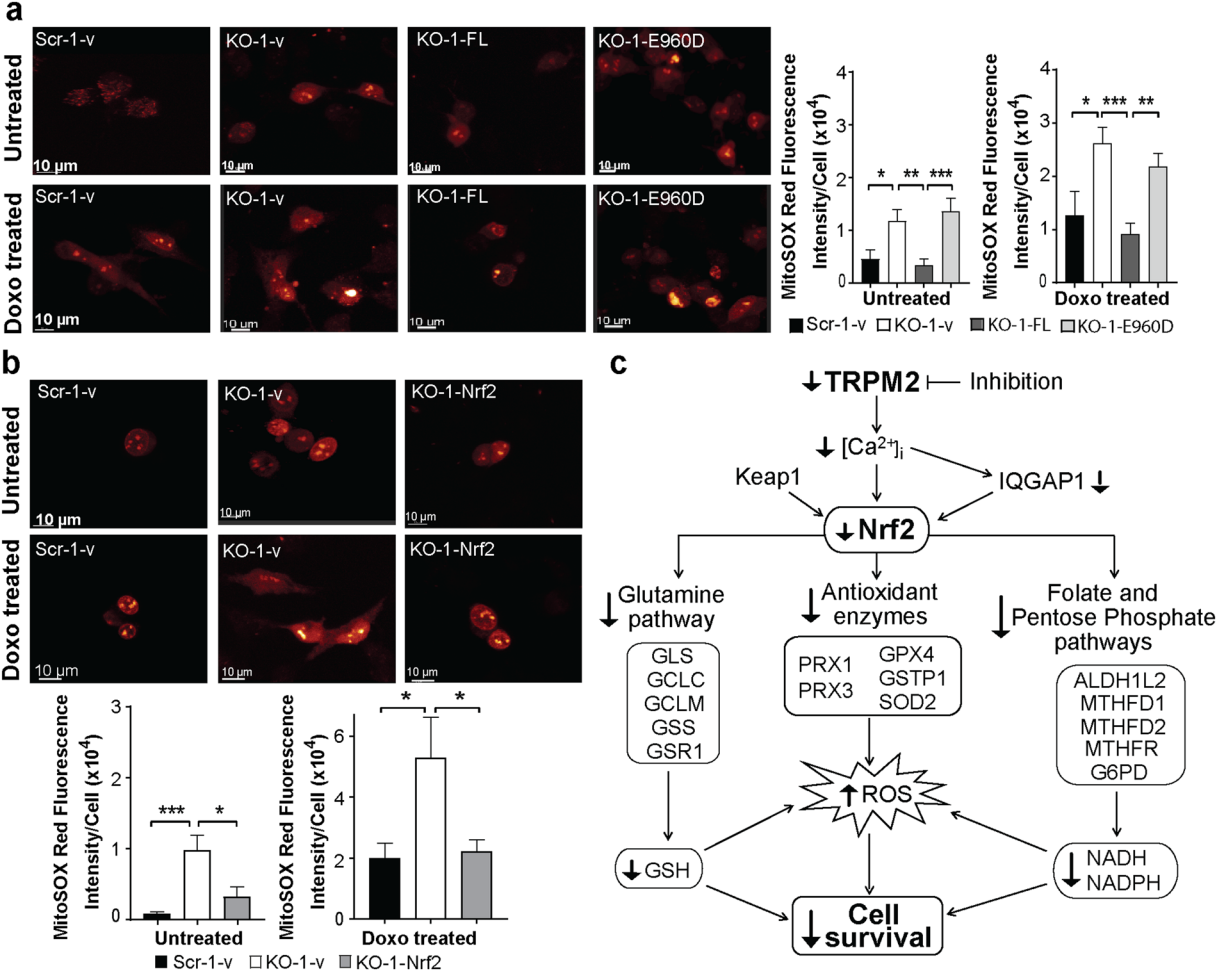
Reconstitution of TRPM2 and Nrf2 modulates ROS in TRPM2 depletion. (**a**) SH-SY5Y cells depleted of TRPM2 and transfected with empty vector (KO-1-v), wild type TRPM2 (KO-1-FL), or the E960D pore mutant (KO-1-E960D) were loaded with MitoSOX Red and studied at baseline and 24 hours after treatment with 0.3 μM doxorubicin. Scrambled control cells were transfected with empty vector (Scr-1-v). Intensity of MitoSOX fluorescence was quantitated with confocal microscopy. Representative fields of untreated or doxorubicin treated cells are shown. Means ± SEM fluorescence intensity of a minimum of 100 cells in at least 10 fields in each group was quantified. Results from a representative experiment of two are shown on the right. (**b**) SH-SY5Y cells depleted of TRPM2 were transfected with empty vector (KO-1-v) or Nrf2 (KO-1-Nrf2). Cells were loaded with MitoSOX Red and studied as described in **a** and results from a representative experiment of three is shown. (**a,b**) *indicates p < 0.05, ** < 0.01, *** < 0.001, one-way ANOVA. (**c**) Schema of impact of TRPM2 inhibition on antioxidant response and ROS levels in neuroblastoma. TRPM2 inhibition or depletion lowers calcium entry. Nrf2 is reduced by down modulation of IQGAP1 and downstream enzymes involved in glutamine/GSH production, antioxidant defense, and folate and the pentose phosphate pathways are decreased. This leads to decreased antioxidant cofactors and impaired antioxidant response, increasing susceptibility to chemotherapeutic agents and decreasing cell survival and tumor growth.

The role of TRPM2 in modulating oxidative stress through Nrf2 was examined by quantitating ROS after TRPM2 or Nrf2 reconstitution in TRPM2 depleted cells. TRPM2 significantly reduced ROS in both untreated and doxorubicin treated TRPM2 depleted cells (Fig. 8a). The pore mutant E960D did not. This demonstrates that an intact calcium pore is critical in regulation of ROS by TRPM2. Expression of Nrf2 also reduced ROS to that in scrambled control cells (Fig. 8b).

## Discussion

Elevated levels of ROS are found in the majority of cancers and promote tumorigenesis through activation of transcription factors and signaling pathways^2^. Increased metabolism which supports accelerated growth contributes to elevated ROS, and cancer cells require increased levels of antioxidants to detoxify ROS and protect viability^4^. When ROS levels rise above a cytotoxic threshold, cells are more susceptible to death^49–51^, and targeting antioxidant defenses has been proposed as a strategy to kill cancer cells^34^. Many cancers including neuroblastoma express high levels of TRPM2, which preserves cell viability by maintaining mitochondrial function, cellular bioenergetics, and modulating ROS^20–23,52^. TRPM2 inhibition has been shown to significantly increase ROS through mechanisms including decreased expression of transcription factors HIF-1/2α and CREB and enzymes involved in the mitochondrial electron transport chain, leading to increased mitochondrial ROS^20,22,53^. Here, TRPM2 is demonstrated to modulate cellular ROS levels by regulating Nrf2, expression of enzymes involved in the antioxidant response downstream of Nrf2, and antioxidant cofactors glutathione, NADPH, and NADH. TRPM2 inhibition enhances death in cancer by impairing cellular antioxidant defenses and increasing ROS to cytotoxic levels.

A major finding here is that TRPM2 function is involved in maintaining GSH, NADPH, and NADH levels. Cells under oxidative stress have a continuous need for regeneration of GSH and NADPH, and when TRPM2 is depleted, these cofactors are significantly reduced, particularly after oxidative stress. GSH is the most abundant antioxidant cofactor in the cell and is utilized by many enzymes which process ROS^54^. NADPH is required to drive glutathione and thioredoxin antioxidant pathways and NADPH–producing enzymes are a rate-controlling step in H_2_O_2_ scavenger flux^54^. Peroxiredoxins (PRXs) and glutathione peroxidases (GPXs) detoxify H_2_O_2_ to water and in the process GSH is oxidized to GSSG. Oxidized GSSG is reduced back to GSH by glutathione reductase (GR), which converts NADPH to NADP^+^. Glutathione transferases are enzymes which conjugate glutathione to toxic electrophilic substances, making them less reactive and consuming GSH in the process. In our experiments, the GSH concentration and ratio of GSH/GSSG were significantly reduced in TRPM2 depleted cells after doxorubicin treatment, indicating reduced conversion of GSSG back to GSH. There was also significant reduction in NADP^+^, NADPH, NAD^+^, and NADH in TRPM2 depletion compared to control cells, and the decrease in NADPH and NADH was greater after oxidative stress. Decreased NADPH may contribute to failure to regenerate GSH from GSSG. Reduction in these key cofactors significantly impairs the antioxidant response.

Another finding is that in TRPM2 depleted cells, expression of the transcription factor Nrf2 is reduced. Nrf2 regulates expression of more than 200 genes involved in antioxidant defense including antioxidant enzymes and those involved in GSH, NADPH, and NADH generation and glutathione metabolism. The decrease in Nrf2 contributes to reduced expression of antioxidant enzymes, lower levels of cofactors and increased ROS. Reduction in antioxidant enzymes included SOD2, which converts superoxide anion to H_2_O_2_ in mitochondria, GPX4, PRX1 and PRX3, which convert H_2_O_2_ to water, and the glutathione transferase GSTP1. In addition, reduced glutathione reductase (GSR1) in the KO results in decreased reduction of GSSG and less regeneration of GSH.

NADPH production, particularly in mitochondria, is critical in maintaining glutathione and other scavengers in a reduced state. Three of the enzymes involved in NADPH/NADH generation were reduced in TRPM2 depleted cells, ALDH1L2, MTHFD1, and MTHFD2^55^. Of these, MTHFD2 is known to be regulated by Nrf2 and MTHFD1 by c-Myc; ALDH1L2 is decreased in TRPM2 depletion by mechanisms unknown at present. Changes in NADPH/NADH regenerating enzymes play a critical role in the decreased NADPH/NADH and the increased ROS found in TRPM2 depletion, and restoration by TRPM2 and Nrf2 expression confirmed the importance of TRPM2 in this pathway.

Nrf2 activation is controlled at multiple levels including transcription, translation, subcellular localization, and through post-translational pathways including Nrf2 stabilization^5^. We did not detect a decrease in Nrf2 mRNA in TRPM2-depleted cells, suggesting that that the reduction in Nrf2 expression was not on a transcriptional basis. To explore other mechanisms through which TRPM2 regulates Nrf2, we examined modulation of Nrf2 stability by two key regulators IQGAP1and Keap1. The third major finding of this work is that the Nrf2 regulatory protein IQGAP1 is reduced in TRPM2 depletion. Nrf2 protein is short-lived and rapidly degraded by the ubiquitin-proteasome pathway, and stabilization alone may enhance nuclear accumulation. IQGAP1 is a GTPase activating protein which interacts with and stabilizes Nrf2 through a calcium dependent pathway^35^. Translocation of Nrf2 and the Nrf2/IQGAP complex into the nucleus increases with intracellular calcium, resulting in enhanced Nrf2 target gene transcription, whereas silencing of IQGAP1 results in decreased Nrf2 and Nrf2 target gene expression^35^. In TRPM2-depleted cells, the decrease in calcium entry following oxidative stress and in IQGAP1 contribute to significantly reduced Nrf2 nuclear protein. The role of calcium entry is shown here because transfection of TRPM2 in the TRPM2 KO restores IQGAP1 and Nrf2 nuclear localization but the calcium pore mutant E960D does not. Of note, higher expression of IQGAP, as seen in TRPM2 expressing cells, has also been reported to contribute to tumor proliferation, invasion, and angiogenesis^56–58^. Mechanisms responsible for the reduction in IQGAP1 mRNA and in c-Myc identified here, and in CREB transcription factor identified previously in TRPM2 depletion, are under investigation. Keap1, a substrate for a Cul3-containing E3 ubiquitin ligase, is a key regulator of Nrf2^36,38^. Under basal conditions, Nrf2 forms a complex with Keap1, promoting Nrf2 polyubiquitination and proteasomal degradation, and maintaining Nrf2 at low levels. Disruption of Keap1-Nrf2 interaction increases stability of Nrf2 and its level. In addition, Keap1 is oxidized in the presence of increased oxidative stress, as occurs in the TRPM2 KO, which decreases association of Keap1 with the Cul3-dependent E3 ubiquitin ligase and reduces Keap1-dependent Nrf2 ubiquitination and degradation^59^. Here, in TRPM2 depleted cells, Keap1 was down regulated, which would be expected to increase Nrf2, but Nrf2 was decreased. This suggests that the decrease in Keap1 was insufficient to enhance Nrf2 levels and other modulating factors such as decreased IQGAP1 predominate. In The RegNetwork: Regulatory Network Repository (www.regnetworkweb.org/), FOXO3 is predicted to regulate Nrf2 expression. FOXO3 is downstream of HIF-1/2α and reduced in TRPM2 depleted cells under oxidative stress^20,22,23^. In addition, Myc regulates both Keap1 and Nrf2 transcription (RegNetwork). However, the decrease in these two transcription factors was not found to reduce Nrf2 mRNA here.

GSH is one of the most important antioxidants in the cell. In TRPM2 depleted cells, glutamine and GSH levels are decreased. The low levels of GSR1 in the KO, resulting in less reduction of GSSG to GSH, contribute to the decrease in GSH. Less GSH generation may also have a role. Glutamine input is the rate limiting step in glutathione synthesis, and glutamine levels are reduced in TRPM2 depleted cells. Glutamine enters the cell through transporters, although many aspects of glutamine transport are unknown^60^. Glutamine transport may not play a role, or the increase in the transporter xCT in TRPM2 depleted cells after doxorubicin may be compensatory for the decrease in glutamine. Both Nrf2 and ATF4 regulate expression of xCT; with suppression of Nrf2 in KO cells, the increase in xCT expression in depleted cells is likely modulated by other transcriptional (ATF4), posttranscriptional, or posttranslational mechanisms^61^. In TRPM2 depletion, the decrease in glutamine and enzymes involved in synthesis of GSH contribute to reduced GSH levels, as well as to reduced ATP and GTP^39,60^. These enzymes include GLS, GCLC, GCLM, GSS, and GSR1 regulated by Nrf2^38,44^ and GLS and GLUD1 by c-Myc^62^.

Decrease in both glutamine and GTP was demonstrated in TRPM2 depletion by metabolomics analysis. Glutamine is a key amino acid which is important in energy generation, is a nitrogen source for synthesis of nucleic acids, participates in cellular redox homeostasis, and has a role in rapid cell proliferation^63^. Glutamine metabolism is a recognized target in cancer therapy^39,64^. Reduced GTP has been reported to impair cancer cell growth by decreasing ribosomal RNA synthesis through inhibition of transcription initiation factor I, which recruits RNA polymerase to the ribosomal DNA promoter^65^. Reduction in GTP has been shown to decrease invasive activity of breast cancer, which was restored by exposure of cells to guanosine or GTP^66^. Decreased levels of these two metabolites by TRPM2 depletion identifies additional pathways through which TRPM2 may modulate cancer progression.

To confirm that off target effects of knockout technology are not responsible for the findings observed in TRPM2 depleted cells, TRPM2 expression was reconstituted. Cell proliferation and viability were restored to that in scrambled control cells by TRPM2 but not the pore mutant. Also, GSH, NAD^+^/NADH, NADP^+^/NADPH, ATP, GTP, glutamine and low ROS levels were restored by TRPM2 but not by constructs with a mutated calcium pore, demonstrating that reduced cofactor levels in TRPM2 depleted cells were mediated through decreased TRPM2-mediated calcium entry. Reconstitution of TRPM2 depleted cells with Nrf2 fully restored GSH and GTP and partially restored cell viability, NADH, NADPH and ATP levels, and reduced ROS. These results demonstrate the important role of Nrf2 in modulation of oxidative stress and survival by TRPM2.

In summary, TRPM2 has a critical role in preserving cell survival after oxidative injury by maintenance of the antioxidant response and cofactors GSH, NADPH, and NADH regulated by Nrf2 (Fig. 8c). Taken together with previous findings, TRPM2 expression promotes cell viability and modulates mitochondrial metabolism, cellular energetics, autophagy, glutamine metabolism and redox balance simultaneously, which make it an intriguing potential drug target in cancer.

## Methods

### Depletion of TRPM2 with CRISPR and generation of stably transfected neuroblastoma cell lines

Generation of TRPM2 CRISPR KO and scrambled control SH-SY5Y cells was described previously^22^. RT-PCR and Western blotting of TRPM2 in neuroblastoma cell lines confirmed TRPM2 depletion. Absence of calcium current in the TRPM2 KO and in the E960D TRPM2 pore mutant in SH-SY5Y cells was reported previously^22^.

### Cell proliferation assay

Transfected cell lines or cells in which TRPM2 was deleted with CRISPR were cultured in media with 250 µg/ml G418 and/or 0.5 µg/ml puromycin. Cell proliferation was assessed by measurement at OD_490nm/690nm_ using XTT (2,3-Bis(2-methoxy-4-nitro-5-sulfophenyl)-2H-tetrazolium-5-carboxanilide) cell proliferation assay (Trevigen Inc., Gaithersburg, MD) following the manufacturer’s instructions^52,67^. In some experiments, cells were treated with doxorubicin (Fresenius, Kabi USA, LLC, Lake Zurich, IL) for specified durations during cell culture.

### GSH, NADP^+^/NADPH, NAD^+^/NADH and ATP quantitation

GSH and GSSG concentrations were measured with the GSH/GSSG-Glo^TM^ assay kit (Promega V6612, Madison, WI). NADP^+^/NADPH levels were quantified using the NADP/NADPH-Glo™ Assay kit (Promega G9082, Madison, WI). NAD^+^/NADH levels were measured with the NAD/NADH-Glo^TM^ assay kit (Promega G0972, Madison, WI). ATP levels were measured with the Cell Titer-Glo Luminescence Cell Viability Assay Kit (Promega, Madison, WI)^22^.

### Immunoblot analysis

Western blotting was performed as described previously^22^ on protein lysates from xenograft tumors and cells cultured *in vitro*. Equivalent amounts of protein were loaded in each lane. Blots were probed with the following antibodies: anti-TRPM2-C (1:1000; Bethyl Laboratories, Montgomery, TX)^68^, anti-ALDH1L2 (1:1000; Abcam, Cambridge, MA), anti-GAPDH (1:10,000; Cell Signaling Technology INC., Danvers, MA), anti-GCLC (1:1000; Abcam), anti-GCLM (1:500; Abcam), anti-GLS (1:500; Abcam), anti-GLUD (1:1,000; Cell Signaling Technology), anti-G6PD (1:1000, Cell Signaling Technology), anti-GSR1 (1:1000; Abcam), anti-GSS (1:500; Santa Cruz Biotechnology, Dallas, TX), anti-GSTP1 (1:3,000; Cell Signaling Technology), anti-GPX4 (1:3000; Abcam), anti-IQGAP1 (1:1000; Abcam), anti-Keap1 (1:1,000; Cell Signaling Technology), anti-MTHFD1 (1:1000; Proteintech, Lower Merion Township, PA), anti-MTHFD2 (1:1,000; Cell Signaling Technology), anti-MTHFR (1:1000; Abcam), anti-c-Myc (1:1000; Abcam), anti-Nrf2 (1:250; Cell Signaling Technology), anti-PRX1 (1:1000; Abcam), anti-PRX3 (1:10,000; Abcam), anti-xCT/SLCA11 (1:1,000; Cell Signaling Technology), anti-SOD2 (1:3000; Abcam), and anti-Actin (1:10,000; Sigma, St. Louis, M). Some experiments were performed with Li-Cor technology and the remainder with Western blotting and ECL. For Western blotting with ECL, blots were washed and incubated with appropriate horseradish peroxidase (HRP)-conjugated antibodies (1:2000; Amersham GE Healthcare, Pittsburgh, PA). Signals were detected with enhanced chemiluminescence (ECL). Intensity of bands was quantitated with densitometry. Some blots were then stripped and reprobed with secondary antibody and ECL after stripping. After it was determined that results were negative, and blots were reprobed with another antibody to measure the total protein. For Li-Cor (Lincoln, NE), proteins on blotted membranes were visualized using secondary antibodies conjugated to IRDye 800CW or IRDye 680RD (Donkey anti-rabbit, 1:20,000, or Donkey anti-mouse, 1:20,000) and the Odyssey CLx fluorescence scanner, according to manufacturer’s instructions^69^. All the bands were analyzed with Image Studio. Images for western blots were prepared with Adobe Illustrator. Because expression was examined for a large number of proteins, a number of the blots were cut before probing around the molecular weights expected for each protein. The full-length probed blots displayed in the figures are shown in Supplemental Fig. 3.

### Subcellular fractionation

Cytosolic and nuclear fractionation was performed using Thermo Scientific (Rockford IL) NE-PER Nuclear and Cytoplasmic Extraction Reagents according to the manufacturer’s protocol.

### Generation of Ca^2+-^impermeable E960D TRPM2 mutant

E960D was created using wild type TRPM2 in pcDNA3.1V5/His vector as a template, Quick Change kit (Stratagene) and the following primers: forward 5′-CTCATCCACAACGACCGCCGGGTGGAC-3′, reverse 5′-TCCACCCGGCGGTCGTTGT GGATGAG-3′. 1–2 μl of 50 μl PCR reaction was used for transformation of competent DH5α (Invitrogen). Clones were verified by digestion of DNA with restriction enzymes and by sequencing.

### Reconstitution of TRPM2 and Nrf2 Function in TRPM2 depleted cells

In TRPM2 reconstitution experiments, SH-SY5Y cells in which TRPM2 was depleted with CRISPR were transfected with wild type TRPM2 subcloned into pcDNA3.1/V5 plasmid, TRPM2 pore mutant E960D subcloned into the same plasmid^28,48,52^, or empty vector using the Neon Transfection System following the manufacturer’s instructions. Scrambled SH-SY5Y control cells were transfected with empty vector. Single cell clones of stably transfected cells were selected with 0.5 µg/ml puromycin and/or 600 μg/ml G418 (Geneticin, an analogue of neomycin; Gemini Bio-Products, West Sacramento, CA) and maintained in culture with 0.5 µg/ml puromycin and/or 250 μg/ml G418 as appropriate. Wild type Nrf2 constructs for reconstitution experiments in the TRPM2 KO were purchased from Addgene (Cambridge, MA), subcloned into pcDNA3.1/V5 plasmid, and transfected as described for TRPM2.

### Measurement of ROS

To measure ROS, SH-SY5Y cells, untreated or treated with doxorubicin (0.3 μM) for 24 hours, were loaded with MitoSOX Red and live cell ROS measurements taken with confocal microscopy (Leica AOBS SP8 laser scanning confocal microscope; Leica Microsystems, Heidelberg, Germany) as described previously^22^.

### Xenograft tumors expressing TRPM2 isoforms

To obtain xenograft tumors for molecular analysis, athymic Nude-FOXn1^nu^ female mice (Harlan Laboratories, Inc, Indianapolis, IN) were injected in one flank with 1.5 × 10^7^ SH-SY5Y cells stably depleted of TRPM2 or control scrambled control cells. Approximately 8–10 mice per group were used in each experiment. Tumor length and width were measured twice weekly over 6–7 weeks. At the completion of each experiment, tumors were harvested, weighed, and frozen for analysis. Significantly reduced proliferation in TRPM2-depleted xenograft tumors compared to control cells was observed as reported previously^22^.

### Metabolomic analysis

Metabolomic analysis was performed using liquid chromatography coupled with mass spectroscopy as described^70^.

### RT-PCR of Nrf2 and IQGAP1

RNA was prepared from neuroblastoma cells using RNeasy kit (Qiagen, Hilden, Germany). cDNA was prepared using the Super Script kit (Invitrogen by Life Technologies). RT-PCR was performed using primers purchased from Applied Biosystems TaqMan@gene Expression assay (NFE2L2-Hs00975961; IQGAP1-Hs008965951; TBP-Hs00427620) and StepOne plus Real Time PCR system (Applied Biosystems). Reactions were run in triplicates. The PCR results were analyzed using Expression Suite software (LifeTechnologies) as relative mRNA level of cycle threshold (CT) value using scrambled CRISPR/cas9 neuroblastoma cell line as a calibrator as described previously^52^.

### Statistics

All results are expressed as mean ± SEM. For analysis of protein expression levels as a function of group (Scr, KO) and doxorubicin exposure time, two-way ANOVA was used. A commercially available software package (JMP Pro 13.0; SAS Institute, Cary, NC) was utilized. For other analyses, Student paired, unpaired T-tests or one-way ANOVA were used as indicated. p < 0.05 was taken as statistically significant unless specified.

### Institutional guidelines

All protocols, procedures, and experiments applied to the mice in this study were approved by the Institutional Animal Care and Use Committee of the Pennsylvania State University College of Medicine. All Methods in this protocol and experiments were performed in accordance with institutional guidelines and regulations and meet Pennsylvania State University College of Medicine Biological Safety and Recombinant DNA requirements.

## Supplementary information

Supplemental Information File

## Data Availability

The data generated during and/or analyzed in the current study are available from the corresponding author on reasonable request.
