# Supplementary material for: The Human Transient Receptor Potential Melastatin 2 Ion Channel Modulates ROS Through Nrf2
Source: Sci Rep. 2019 Oct 1;9:14132. doi: 10.1038/s41598-019-50661-8 (PMC6773863; doi:10.1038/s41598-019-50661-8)

Supplemental Information File

The Human Transient Receptor Potential Melastatin 2 Ion Channel Modulates ROS through Nrf2

Lei Bao[1], Fernanda Festa[1,2], Christopher S. Freet[1], John P. Lee[1], Iwona M. Hirschler-Laszkiewicz[1], Shu-jen Chen[1], Kerry A. Keefer[1], Hong-Gang Wang[1,3], Andrew D. Patterson[4], Joseph Y. Cheung[5,6], and Barbara A. Miller[1,2*]

Affiliations: Departments of [1]Pediatrics, [2]Biochemistry and Molecular Biology, and [3]Phamacology, The Pennsylvania State University College of Medicine, P.O. Box 850, Hershey, Pennsylvania17033, USA

Department of [4]Molecular Toxicology, The Pennsylvania State University, University Park, Pennsylvania

[5]The Center of Translational Medicine and the Department of [6]Medicine, Lewis Katz School of Medicine, Temple University, Philadelphia, Pennsylvania 19140

Supplementary Figure S1

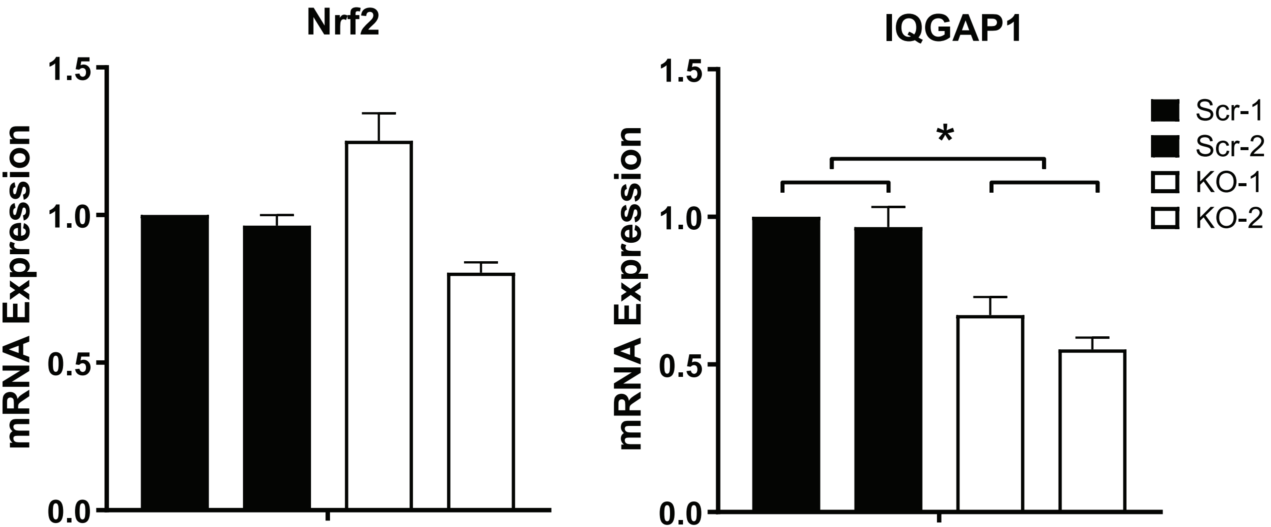

Supplementary Figure S1. RT-PCR of Nrf2 and IQGAP1. RT-PCR was used to measure Nrf2 and IQGAP1 mRNA in TRPM2 depleted SH-SY5Y cells. Results in the KOs were normalized to the average of the scrambled controls in each experiment, and the means $\pm$ SEM for five experiments are shown. *$p<0.0001$, Student's T-test.

Supplementary Figure S2

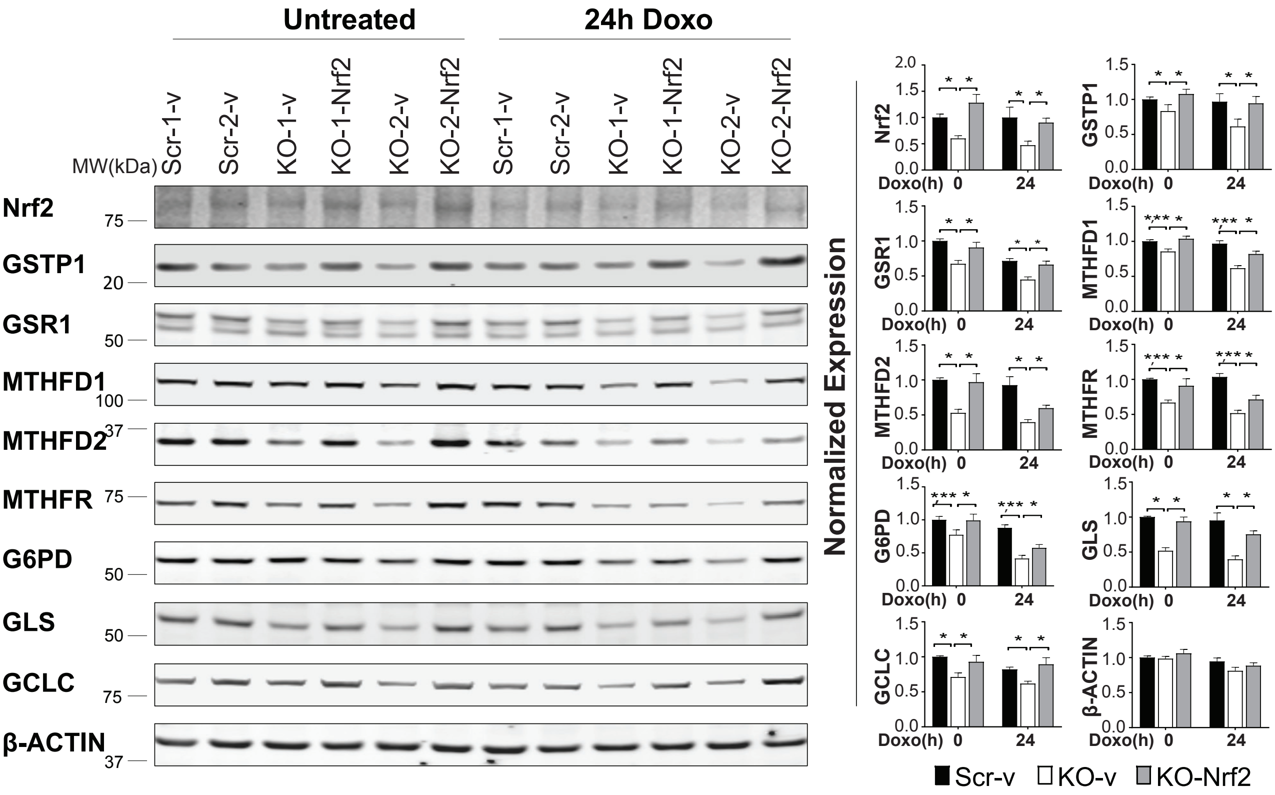

Supplementary Figure S2. Expression of Nrf2 fully restores GLS and GCLC and partially restores expression of other antioxidant enzymes in TRPM2 depleted cells. SH-SY5Y cells in which TRPM2 was depleted were stably transfected with empty vector (2 clones, KO-1-v and KO-2-v) or Nrf2 (2 clones, KO-1-Nrf2 and KO-2-Nrf2). Scrambled control cells were transfected with empty vector (2 clones, Scr-1-v and Scr-2-v). Cells were untreated or treated with 0.3 μM doxorubicin for 24 hours. Western blotting was performed with antibodies to Nrf2, GSTP1, GSR1, MTHFD1, MTHFD2, MTHFR, G6PD, GLS, GCLC, and actin. Representative blots from three experiments are shown. Intensity of bands was quantitated with Li-Cor technology. Blots were normalized by comparing bands to each protein's time 0 average scrambled control. Normalized means $\pm$ SEM for Scr and KO cells at each time point from three experiments are shown on the right. *$p \leq 0.02$ group effect; **$p \leq 0.05$, group x doxorubicin exposure time interaction effect; two-way ANOVA.

Supplementary Figure S3

These figures show non-cropped Western blot images used to create Figures 2,3,4,6, and Supplementary Figure S2. Blots were cut around the expected molecular weights for each protein before probing, and the probed blots are shown. Red rectangles mark the bands used.

Full-Length Blots Figure 2a

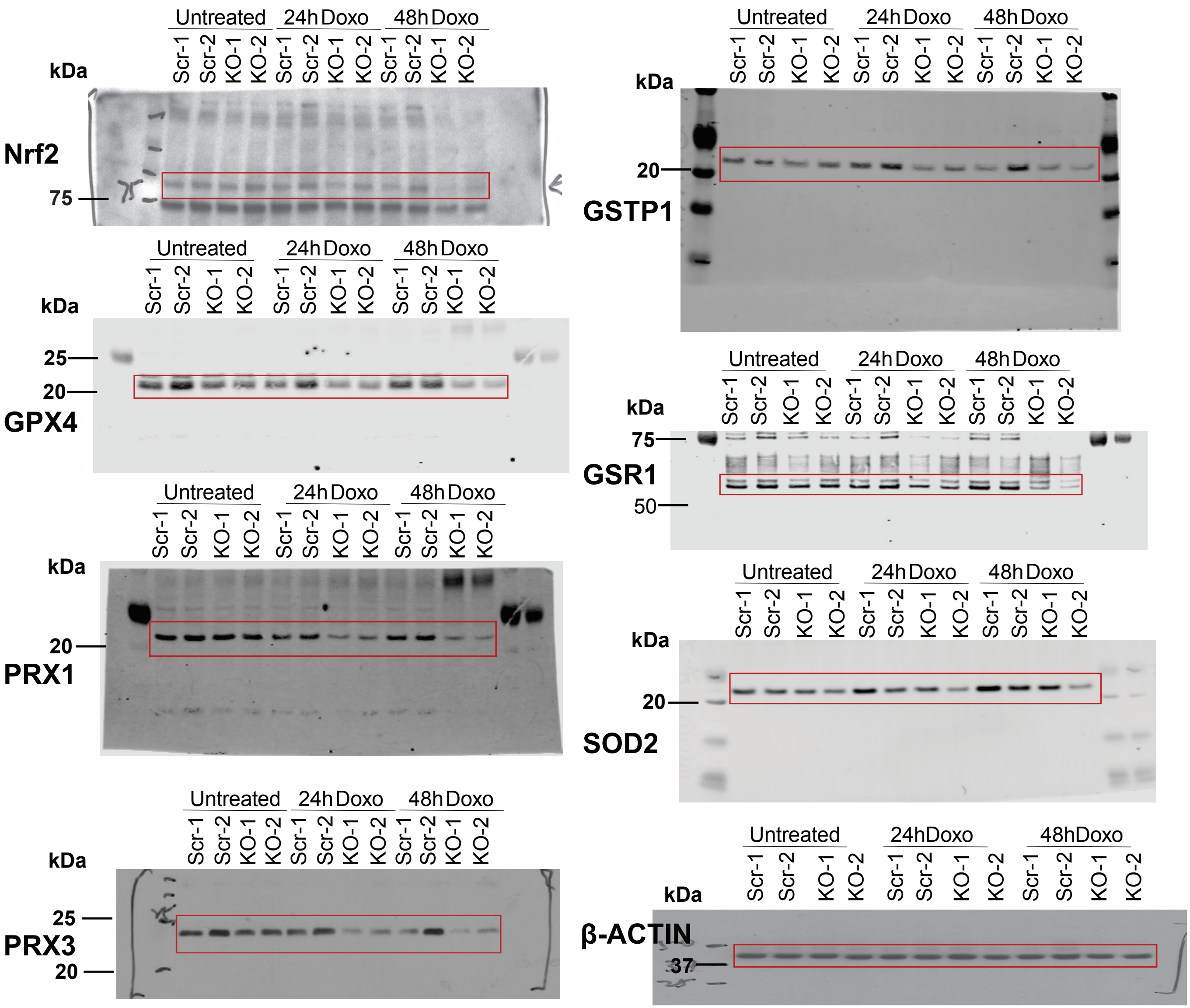

Supplementary Figure S3 (continued)

Full-Length Blots Figure 2b

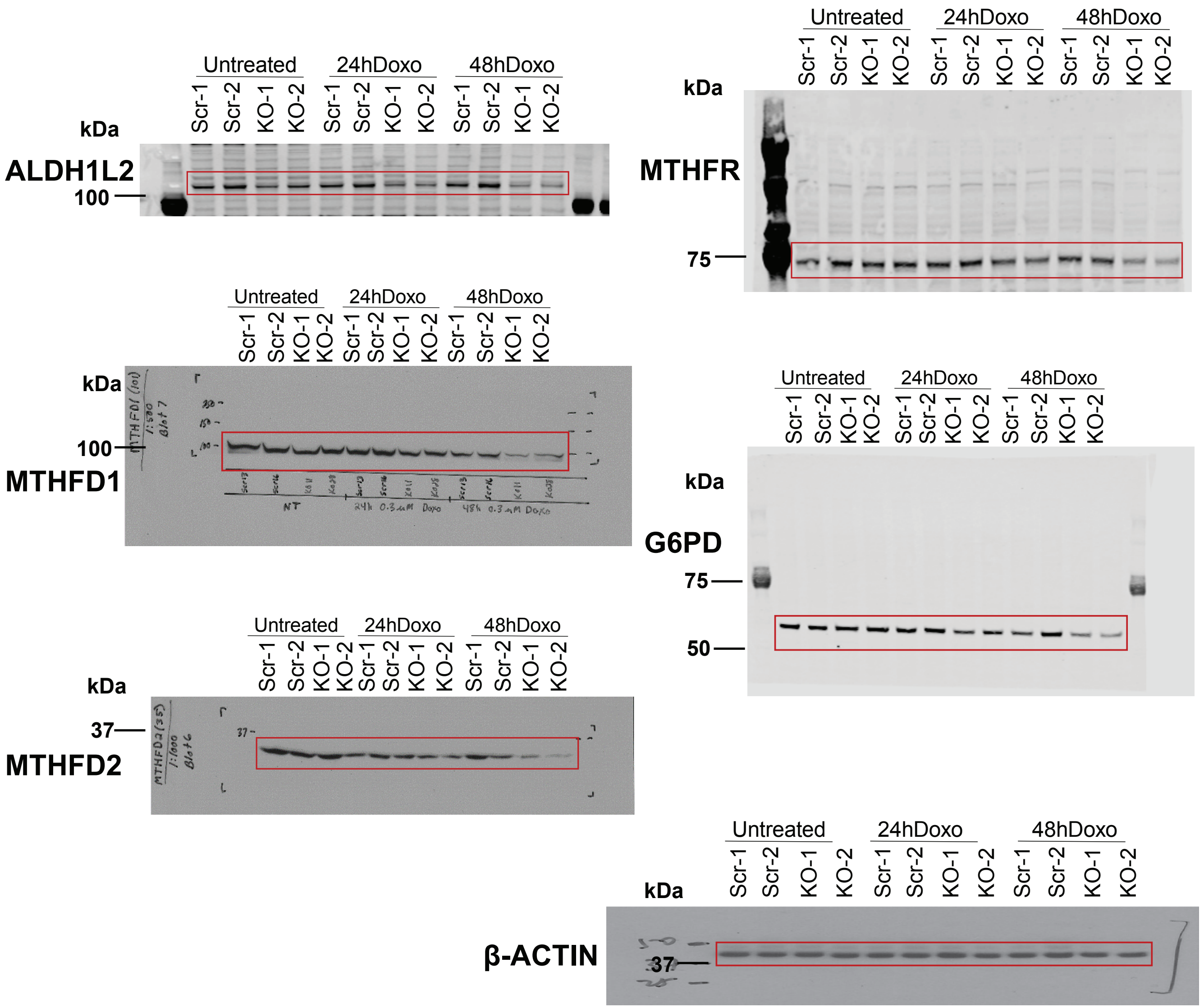

Supplementary Figure S3 (continued)

Full-Length Blots Figure 2c

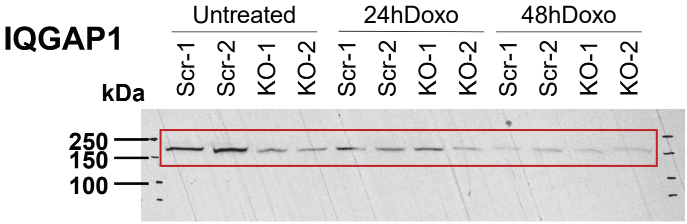

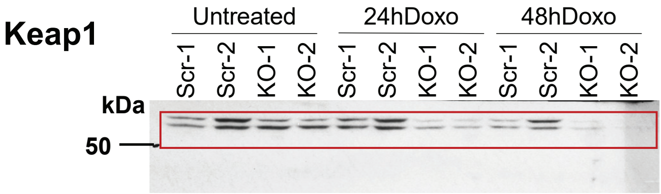

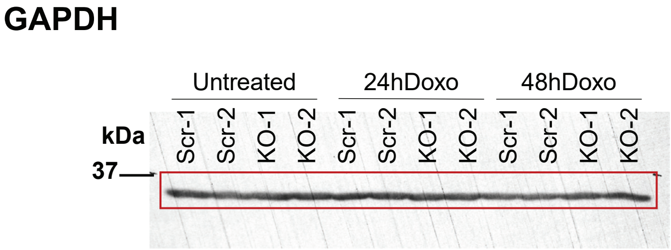

Supplementary Figure S3 (continued)

Full-Length Blots Figure 3a

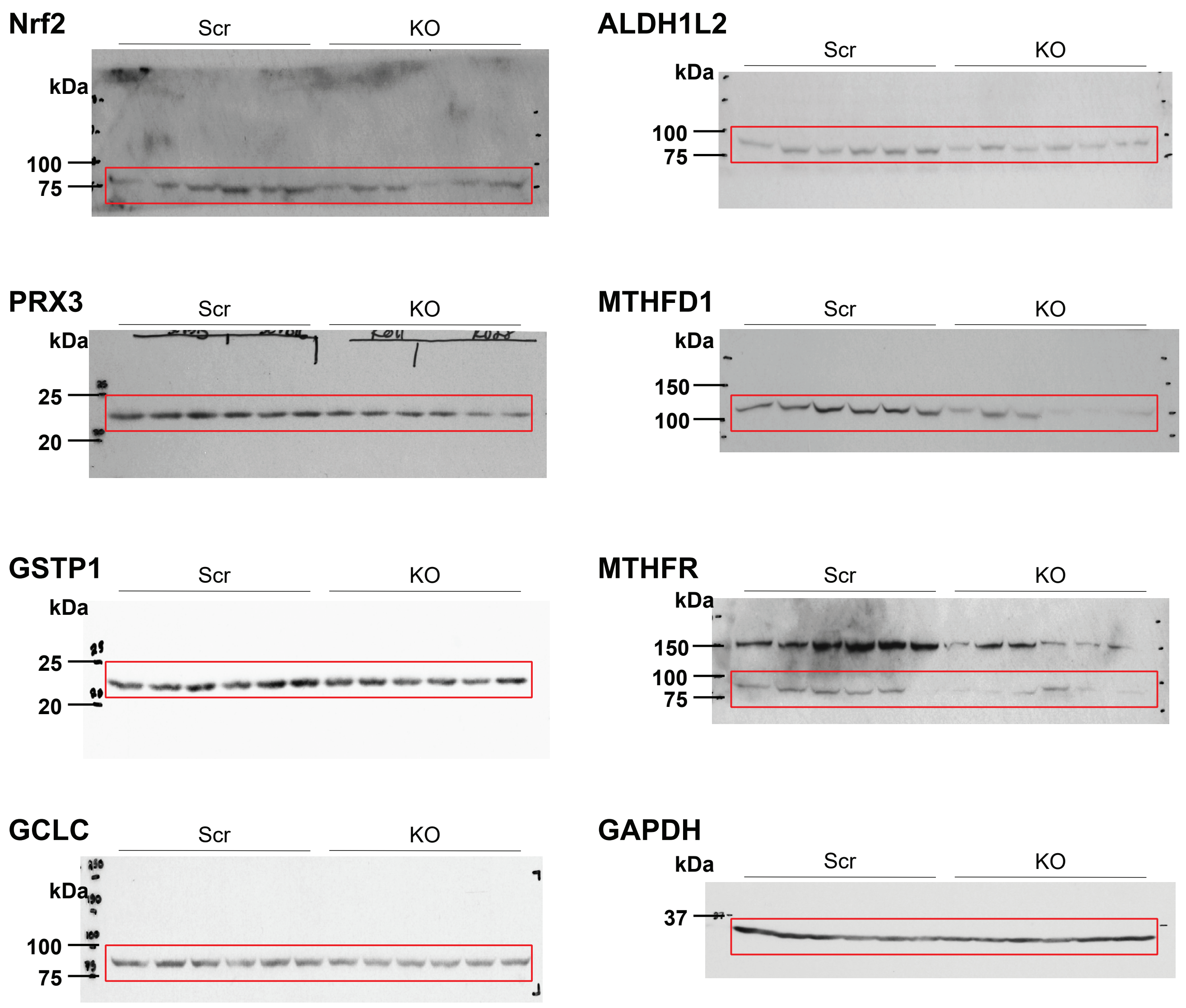

Supplementary Figure S3 (continued)

Full-Length Blots Figure 3b

IQGAP1

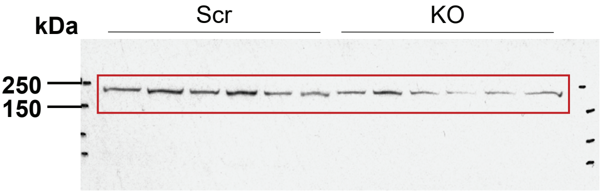

Keap1

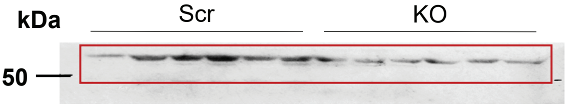

GAPDH

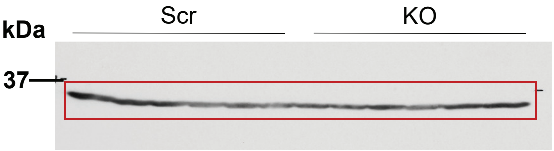

Supplementary Figure S3 (continued)

Full-Length Blots Figure 4e

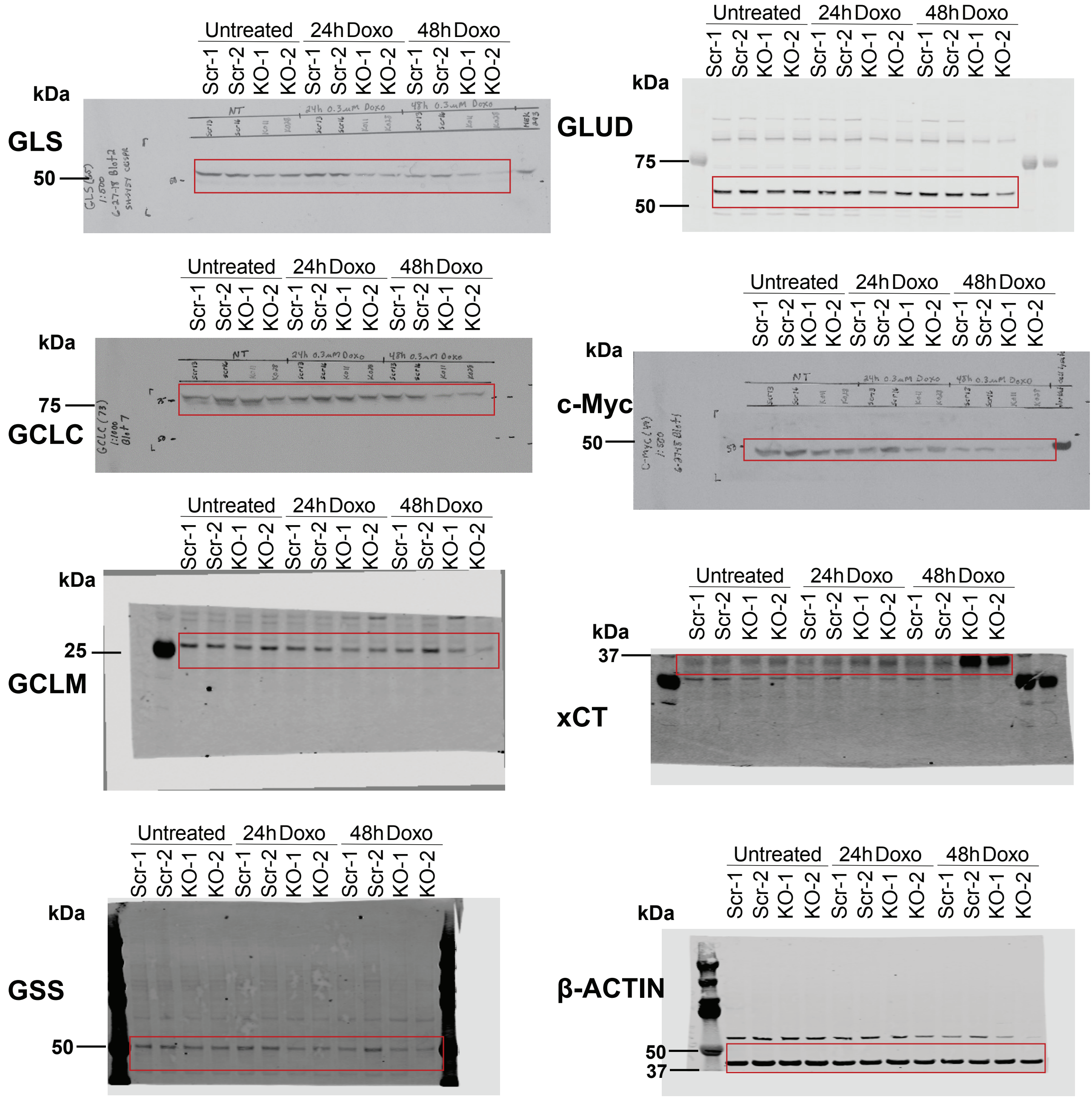

Supplementary Figure S3 (continued)

Full-Length Blots Figure 6a

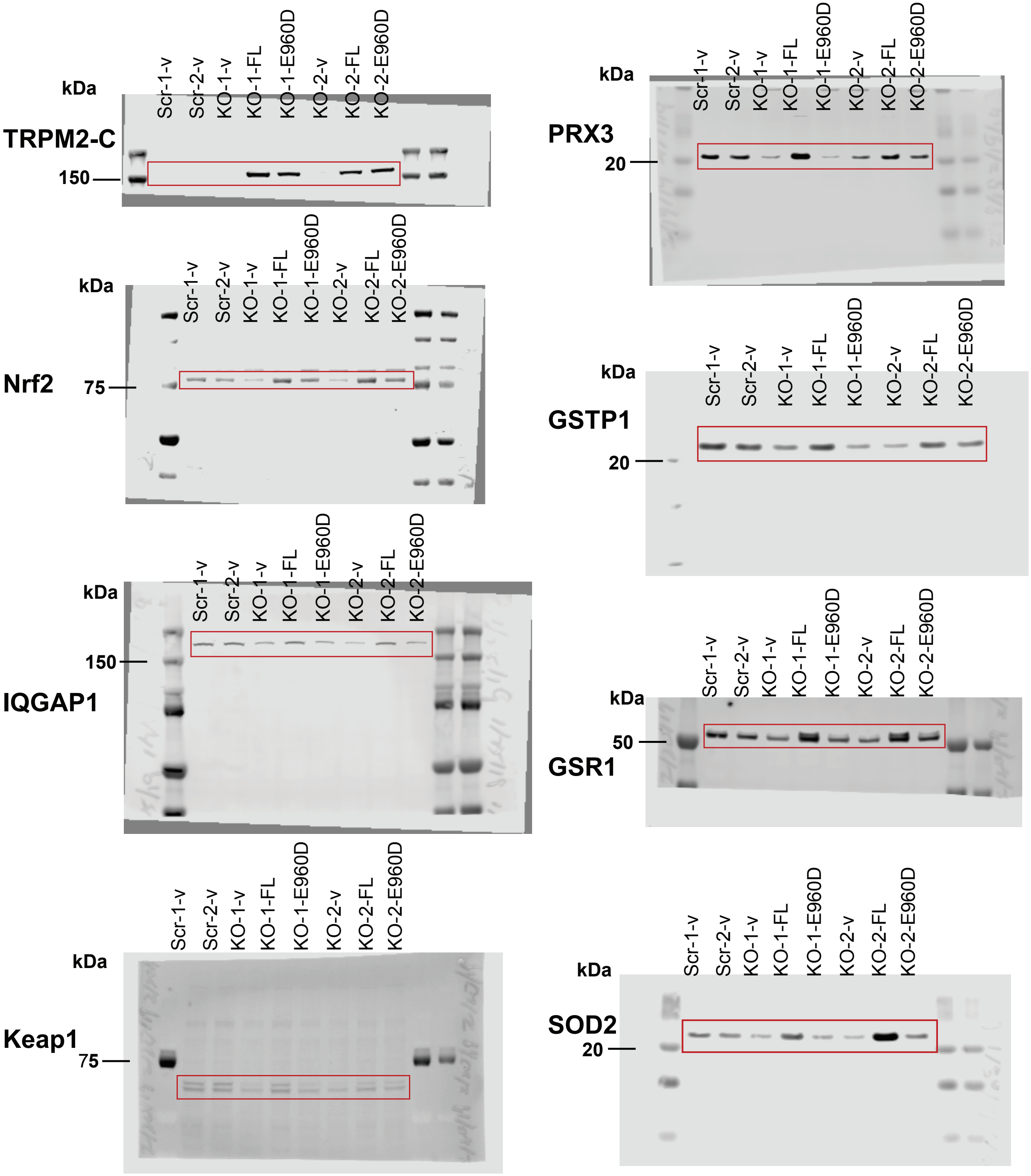

Supplementary Figure S3 (continued)

Full-Length Blots Figure 6a (continued)

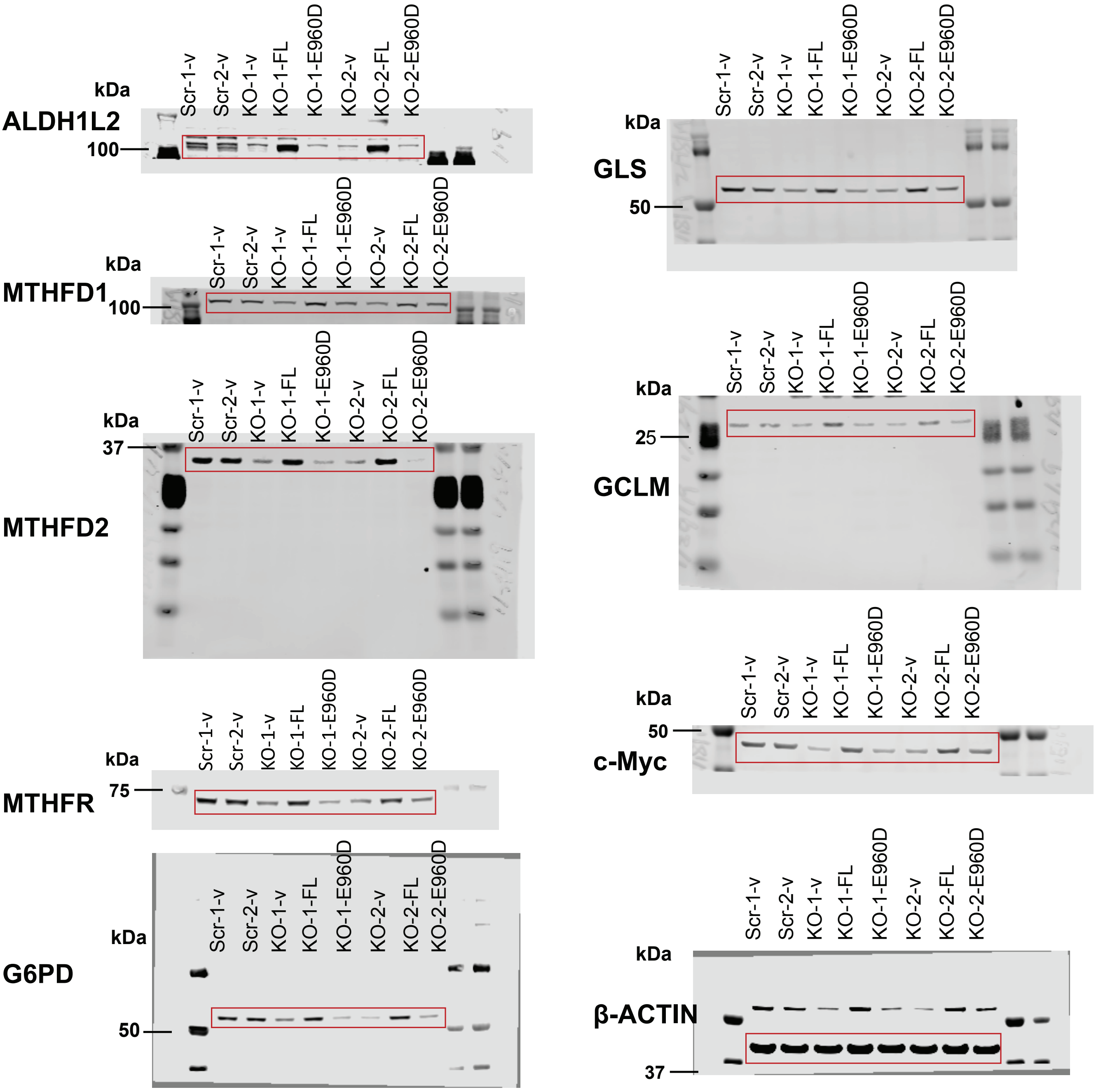

Supplementary Figure S3 (continued)

Full-Length Blots Figure 6b

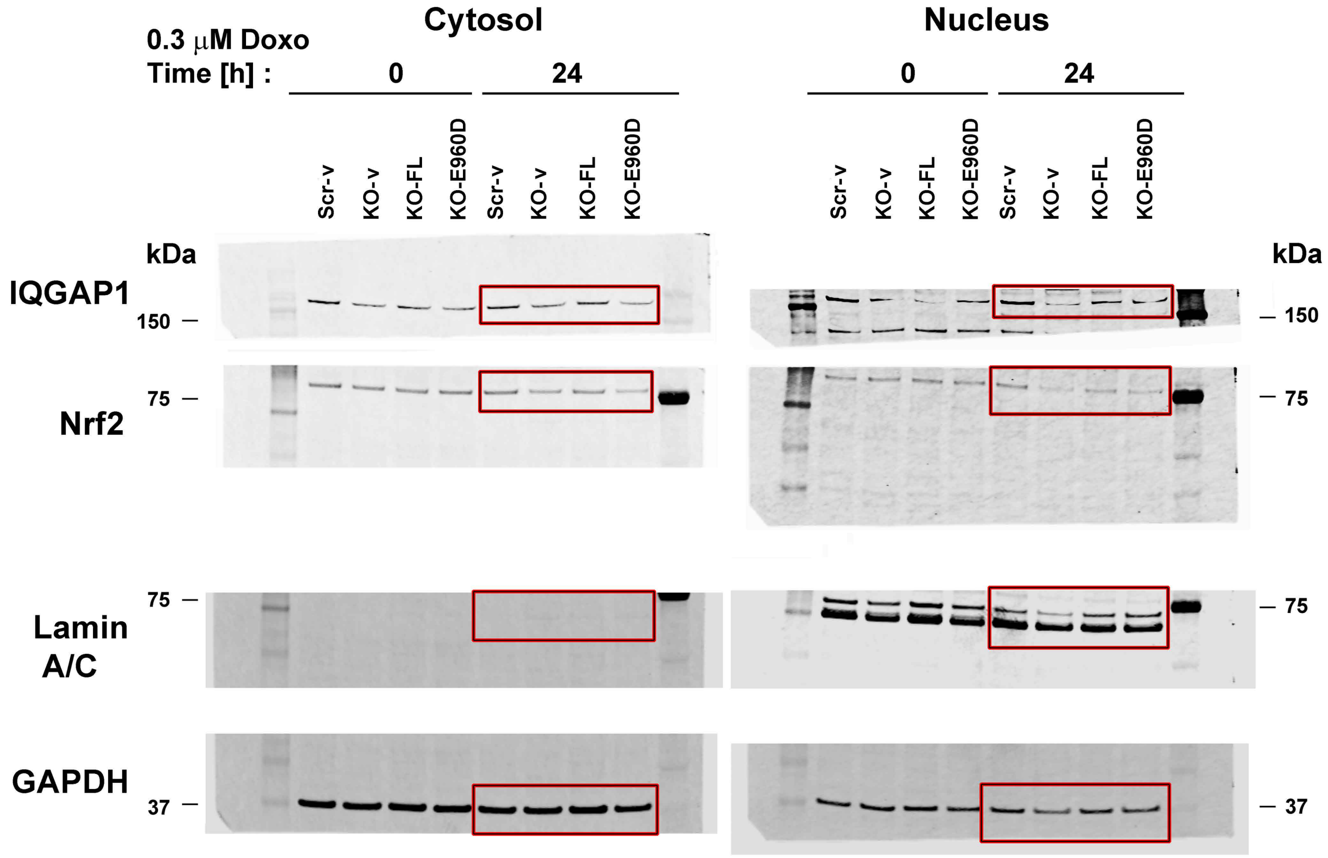

Supplementary Figure S3 (continued)

Full-Length Blots Figure Supplementary S2

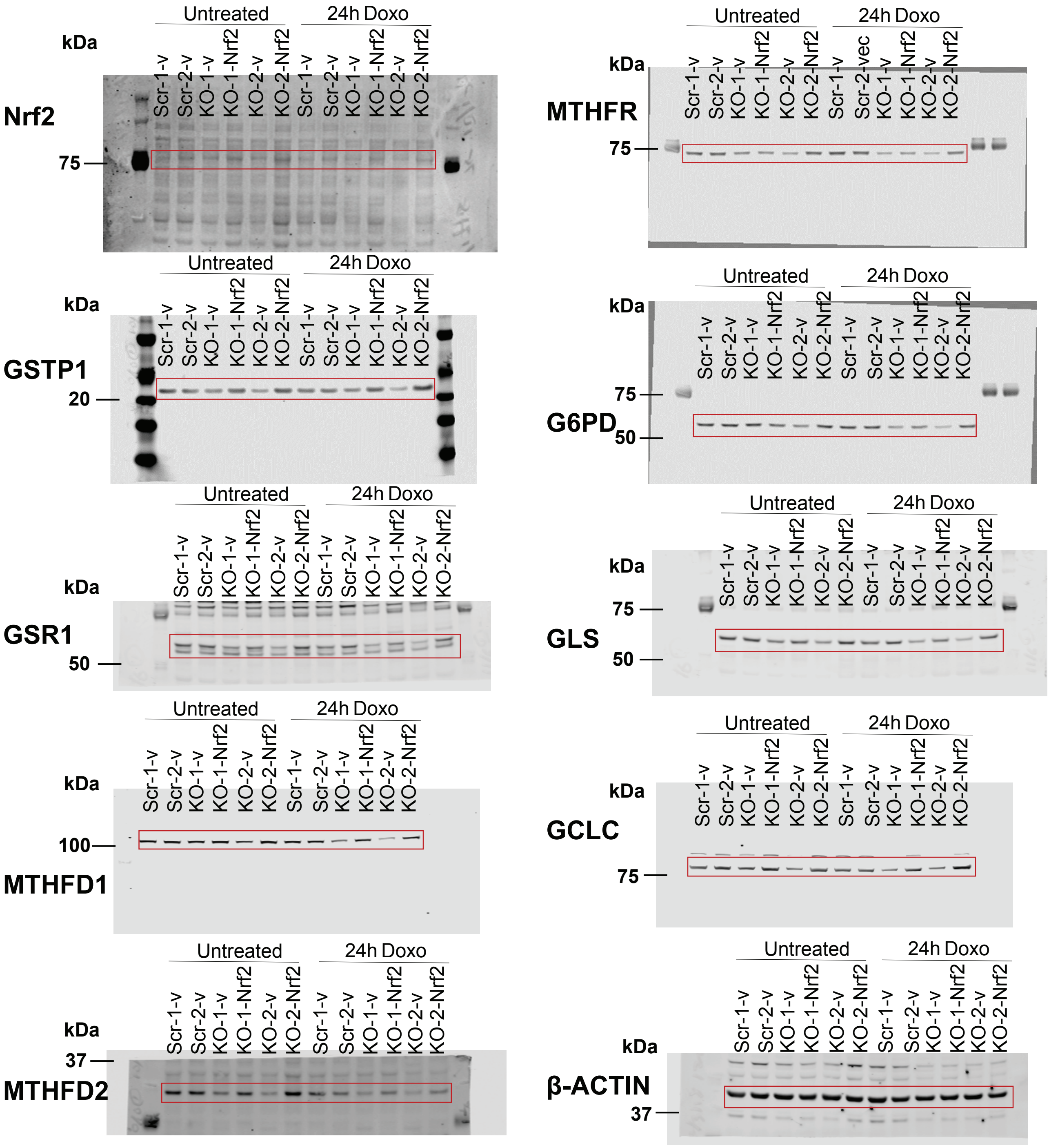

Supplement: Supplementary file 1 — Supplemental Information File [file 41598_2019_50661_MOESM1_ESM.pdf]
